# Automated treatment planning of prostate stereotactic body radiotherapy with focal boosting on a fast‐rotating O‐ring linac: Plan quality comparison with C‐arm linacs

**DOI:** 10.1002/acm2.13345

**Published:** 2021-07-28

**Authors:** Robin De Roover, Wouter Crijns, Kenneth Poels, Bertrand Dewit, Cédric Draulans, Karin Haustermans, Tom Depuydt

**Affiliations:** ^1^ Department of Radiation Oncology University Hospitals Leuven Leuven Belgium; ^2^ Department of Oncology KU Leuven Leuven Belgium

**Keywords:** automation, focal boost, O‐ring linac, plan quality, prostate cancer, SBRT

## Abstract

**Purpose:**

The integration of auto‐segmentation and automated treatment planning methods on a fast‐rotating O‐ring linac may improve the time efficiency of online adaptive radiotherapy workflows. This study investigates whether automated treatment planning of prostate SBRT with focal boosting on the O‐ring linac could generate plans that are of similar quality as those obtained through manual planning on clinical C‐arm linacs.

**Methods:**

For 20 men with prostate cancer, reference treatment plans were generated on a TrueBeam STx C‐arm linac with HD120 MLC and a TrueBeam C‐arm linac with Millennium 120 MLC using 6 MV flattened dual arc VMAT. Manual planning on the Halcyon fast‐rotating O‐ring linac was performed using 6 MV FFF dual arc VMAT (HA2‐DL10) and triple arc VMAT (HA3‐DL10) to investigate the performance of the dual‐layer MLC system. Automated planning was performed for triple arc VMAT on the Halcyon linac (ET3‐DL10) using the automated planning algorithms of Ethos Treatment Planning. The prescribed dose was 35 Gy to the prostate and 30 Gy to the seminal vesicles in five fractions. The iso‐toxic focal boost to the intraprostatic tumor nodule(s) was aimed to receive up to 50 Gy. Plan deliverability was verified using portal image dosimetry measurements.

**Results:**

Compared to the C‐arm linacs, ET3‐DL10 shows increased seminal vesicles PTV coverage (D_99%_) and reduced high‐dose spillage to the bladder (V_37Gy_) and urethra (D_0.035cc_) but this came at the cost of increased high‐dose spillage to the rectum (V_38Gy_) and a higher intermediate dose spillage (D2cm). No statistically significant differences were found when benchmarking HA2‐DL10 and HA3‐DL10 with the C‐arm linacs. All plans passed the patient‐specific QA tolerance limit.

**Conclusions:**

Automated planning of prostate SBRT with focal boosting on the fast‐rotating O‐ring linac is feasible and achieves similar plan quality as those obtained on clinical C‐arm linacs using manual planning.

## 1 INTRODUCTION

External beam radiotherapy (EBRT) is one of the standard treatment options for men with localized prostate cancer (PCa).^1^ Continuous improvement and development of radiotherapy (RT) techniques allowed for the physical dose escalation of the target volume, improving biochemical disease‐free, distant metastases‐free, and even overall survival in patients with high‐risk PCa.^2^, ^3^, ^4^ However, by escalating the dose to the entire prostate gland these positive results come at the expense of increased toxicity. Since most local recurrences occur at the location of the primary tumor,^5^ adding a precise focal boost to the intraprostatic lesions may improve disease control while obtaining a more favorable toxicity profile compared to whole‐gland dose‐escalation.^6^, ^7^ The benefit of focal boosting has recently been demonstrated by the primary endpoint analysis of the phase III FLAME trial, which showed improved biochemical disease‐free survival at 5‐year follow‐up when adding a focal boost.^8^ Today, this focal boosting strategy is also under investigation in multiple phase I/II trials using stereotactic body radiotherapy (SBRT), such as the phase II hypo‐FLAME study.^9^ These ultra‐hypofractionated treatments have been demonstrated to provide good disease control with minimal toxicity and are more convenient for the patient.^10^, ^11^, ^12^, ^13^, ^14^ The combination of SBRT with a simultaneous integrated focal boost, however, results in a complex heterogeneous dose distribution with steep dose gradients both inside and outside the prostate. Given the low number of treatment fractions (≤7 fractions) to deliver this complex dose distribution, accurate treatment delivery is crucial.

The current standard practice in image‐guided radiotherapy (IGRT) is to perform daily target position verification and online repositioning to account for the interfractional prostate movement.^15^, ^16^ However, most online repositioning methods can only partially correct for rotation shifts, as the magnitude of lateral rotations often exceeds the maximum possible pitch correction of the treatment couch.^17^, ^18^ Moreover, these methods generally ignore prostate deformations and the independent movement of the prostate and its surrounding organs at risk (OAR). The significance of these residual prostate rotations and deformations increases within the context of SBRT with focal boosting due to the localized dose escalation and the tighter than conventional margins that are used.^19^, ^20^, ^21^ In addition, parts of the rectum and bladder could move into the high dose region potentially increasing treatment‐related toxicity. Online treatment plan adaptation could account for these interfractional anatomy variations but is challenging since all of the treatment planning steps (i.e., contouring, plan optimization, and quality assurance) must be reperformed while the patient is lying on the treatment couch.^22^, ^23^ It is, therefore, important that the entire adaptation procedure does not prolong the treatment fraction considerably as this might increase the risk of intrafraction motion and can become uncomfortable for the patient.^24^

The integration and automation of the different steps in the adaptation process may improve the time efficiency of online adaptive radiotherapy (ART). The present work is a pilot study in the preparation of the implementation of a new treatment platform, Ethos Therapy (Varian Medical Systems, Palo Alto, CA), which integrates an artificial intelligence‐driven automated adaptive workflow on a fast‐rotating O‐ring linac system, to perform an online treatment plan adaptation for prostate SBRT with focal boosting. Briefly, this treatment platform uses iterative cone‐beam computed tomography (iCBCT) imaging^25^, ^26^ and auto‐segmentation algorithms to visualize and delineate the anatomy of the day. Based on these contours, a new plan of the day is optimized for today's anatomy and compared with the recalculated dose distribution of the original treatment plan to evaluate which plan is most suitable. Treatment delivery is performed using either sliding‐window intensity‐modulated radiotherapy (IMRT) or volumetric modulated arc therapy (VMAT) on a fast‐rotating O‐ring linac system, Halcyon (Varian Medical Systems). This linac system combines a 6 MV flattening filter‐free (FFF) beam with a dual‐layer multi‐leaf collimator (MLC) system mounted on an O‐ring gantry. All MLC leaves have a leaf width of 10 mm and the upper leaves are offset by 5 mm with respect to the lower leaves.^27^

The automation of the treatment planning process, however, changes some of the typical steps in the conventional treatment planning workflow. Most importantly, the actual optimization process is completely handled by an automated planning algorithm that initiates, controls, and monitors the optimization process. Once the inputs have been provided, the entire optimization process is fixed and it is no longer possible for the planner to intervene nor to monitor this process. This input is provided in terms of a prioritized list of clinical goals that describe the planning objectives and their trade‐offs. When the plan generation is initiated, these clinical goals will be converted into a set of optimization objectives that drive the optimization process. The relative priority of the clinical goals also determines the order in which the optimization objectives are manipulated by the automated planning algorithm, with objectives being modified such that failures in higher‐priority goals are worked on before lower‐priority goals. This approach differs distinctively from conventional manual treatment planning in which the planner determines the optimization objectives and their weights explicitly and is able to monitor and modify these throughout the optimization process to obtain a dose distribution that meets the clinical expectations. As a consequence, the main focus in automated treatment planning is shifted toward the initial translation of the planning objectives into a prioritized list of clinical goals as this directs the remaining steps in the process. Moreover, during treatment plan adaptation the same prioritized list will be used together with the automated planning algorithm to optimize the plan for the anatomy of the day.

As a first step in the implementation of this treatment platform, this study investigates whether automated treatment planning of prostate SBRT with focal boosting on the fast‐rotating O‐ring linac is able to generate treatment plans of similar quality as those obtained on standard clinical C‐arm linacs. To this end, reference treatment plans were generated on a C‐arm linac with high‐resolution MLC (2.5 mm leaf width) and a C‐arm linac with standard resolution MLC (5 mm leaf width). Next, treatment plans were generated on the fast‐rotating O‐ring linac using manual treatment planning to investigate the influence of the dual‐layer MLC system on the achievable plan quality. Finally, treatment plans were generated on the fast‐rotating O‐ring linac using the automated treatment planning algorithms of Ethos Treatment Planning and the obtained plan quality was benchmarked with those achieved through manual planning on the investigated linac systems.

## 2 MATERIALS AND METHODS

### 2.1 Patient population

Computed tomography (CT) with registered multiparametric magnetic resonance imaging (mpMRI) (including T2 weighted (T2w), diffusion‐weighted imaging (DWI), and dynamic contrast‐enhanced (DCE) sequences) data of 20 men with intermediate or high‐risk PCa were used for this treatment planning study. All these patients were previously treated on the FLAME trial (NCT01168479) (4 out of 20 patients) or the hypo‐FLAME trial (NCT02853110) (16 out of 20 patients) in a single institution and at least one tumor needed to be visible on mpMRI for study inclusion. Patient and tumor characteristics are presented in Table 1. Patients were planned and treated in the supine position using a knee wedge and foot block to assist patient positioning. Patients were asked to have a comfortably full bladder and empty their bowel or use micro enema prior to simulation. The imaging procedures and scanning parameters are presented in Supplemental Table S1. The whole prostate gland was delineated according to the ESTRO ACROP consensus guideline.^28^ The intraprostatic lesion(s) (GTV_boost_) were delineated on mpMRI.^29^, ^30^ The prostate CTV (CTV_prostate_) included the whole prostate gland and a 4 mm isotropic margin around the GTV_boost_, excluding organs at risk (OAR). In four patients, two separate GTVs were contoured. One patient had three separate GTVs contoured. In total, 26 separate GTVs were contoured with volumes ranging from 0.10 to 6.21 cc. According to the discretion of the treating physician, for all patients, a second seminal vesicle CTV (CTV_SV_) was created.^31^ OARs were delineated based on the RTOG consensus guideline.^32^ All delineations were performed by a trained radiation oncologist and contouring of the focal boost region on mpMRI was supervised by a radiologist experienced in uro‐oncology. Planning target volumes (PTV) were created by expanding the CTVs using an isotropic 4 mm margin. The target and OAR volumes are presented in Table 1. The isocenter was positioned at the prostate PTV centroid.

**TABLE 1:** Patient and tumor characteristics

Characteristic	Number or median (range)
Age [year]	76 (67, 80)
Initial PSA [ng/mL]	10.18 (1.79, 21.46)
EAU risk group	
Low risk	0
Intermediate risk	7
High risk	13
Clinical tumor stage	
cT2a	7
cT2b	2
cT3a	11
Nodal stage	
cNx	18
pN0 (<10 LN removed)	0
pN0 (≥10 LN removed)	2
ISUP grade group	
1	0
2	4
3	10
4	2
5	4
Number of lesions	
1	15
2	4
3	1
Volume [cc]	
GTV_boost_	0.69 (0.10, 6.21)
Prostate	30.83 (17.85, 73.15)
Seminal vesicles	8.92 (2.36, 18.83)
PTV_prostate_	62.38 (38.98, 134.48)
PTV_SV_	26.00 (7.69, 45.66)
Rectum	45.51 (34.96, 119.07)
Bladder	98.79 (38.79, 198.12)

Note

The number or median and range (min, max) over all 20 patients are given for each characteristic.

### 2.2 Planning objectives

Target volume dose prescriptions and OAR dose constraints were based on the hypo‐FLAME trial,^16^ and are depicted in Table 2. The prescribed dose was 35 Gy to the PTV_prostate_ and 30 Gy to the PTV_SV_ in five fractions. PTV objectives were such that the PTV_prostate_ V_33.25 Gy_ ≥99% and the PTV_SV_ V_30 Gy_ ≥99%. The objectives for GTV_boost_ aimed to deliver a minimum total dose of 40 Gy on at least 99% of the volume and up to 50 Gy as long as the OAR sparing was not at risk, that is, iso‐toxic boosting. An isotropic planning organ at risk volume (PRV) margin of 2 mm surrounding the rectum (PRV_rectum_) and urethra (PRV_urethra_) were used as a high‐dose avoidance zone with a maximum dose constraint of 42 Gy.

**TABLE 2:** Target volume dose prescriptions and organs at risk (OAR) dose constraints

Structure	Volume	Expected dose
Target coverage		
GTV_boost_	≥99%	40 Gy; aimed up to 50 Gy^*^
	0.1 cc	≤52 Gy (if possible)
CTV_prostate_	≥99%	35 Gy
PTV_prostate_	≥99%	33.25 Gy
CTV_SV_	≥99%	30 Gy
PTV_SV_	≥99%	30 Gy
OAR constraints		
Rectum	0.035 cc (=D_max_)	40 Gy
	≤1 cc	38 Gy
	≤2 cc (if possible <1 cc)	35 Gy
	≤15%	32 Gy
	≤20%	28 Gy
	≤ 50%	23.5 Gy
	≤70%	20.5 Gy
	≤75%	17 Gy
PRV_rectum_	0.035 cc (=D_max_)	≤42 Gy
Bladder	≤1 cc	42 Gy
	≤5 cc	37 Gy
	≤15%	32 Gy
	≤20%	28 Gy
Urethra	0.035 cc (=D_max_)	≤42 Gy
PRV_urethra_	0.035 cc (=D_max_)	≤ 2 Gy
Anal canal	≤1 cc	37.85 Gy
	≤40%	21.4 Gy
	≤60%	19.2 Gy
Penile bulb	≤90%	20 Gy
Femoral head and neck	≤5%	28 Gy

Note

Target volume dose prescriptions and OAR dose constraints are based on the hypo‐FLAME clinical trial.^16^

CTV, clinical target volume; PTV, planning target volume; GTV, gross tumor volume; PRV, planning organ at risk volume; D_max_, maximum dose.

^*^ GTV_boost_ was aimed to receive up to 50 Gy, as long as the OAR dose constraints were not exceeded.

### 2.3 Linac systems

The Halcyon system consists of a single‐energy 6MV FFF straight‐through linac mounted on an O‐ring gantry. Its encapsulated O‐ring design allows for a maximum gantry rotation speed of 4 rpm during imaging and 2 rpm during treatment delivery. The jawless dual‐layer MLC is composed of 29 proximal leaf pairs (upper bank) and 28 distal leaf pairs (lower bank). Two additional distal leaf pairs outline the maximum field size (28 × 28 cm² at isocenter). All MLC leaves have a projected leaf width of 10 mm at the isocenter and the proximal leaves are offset by 5 mm from the distal leaves. The maximum leaf speed is 5 cm/s and the maximum leaf span is 28 cm. The absolute dose output was calibrated as 1 cGy/MU delivered to water at 10 depth and 90 cm source‐surface distance with a 10 × 10 cm² field size.^33^

For plan quality comparison, a TrueBeam STx C‐arm linac (Varian Medical Systems) with a high‐resolution HD120 MLC and TrueBeam C‐arm linac (Varian Medical Systems) with a standard resolution Millennium 120 MLC were used. The HD120 MLC consists of 32 central leaf pairs of 2.5 mm width and 28 outer leaf pairs of 5 mm width whereas the Millennium 120 MLC consists of 40 central leaf pairs of 5 mm width and 20 outer leaf pairs of 10 mm width. The leaves of both MLCs are mounted on opposing, movable carriages and have a maximum leaf span of 15 cm. Both MLCs have a maximum leaf speed of 2.5 cm/s. The maximum gantry rotation speed of both C‐arm linacs is 1 rpm. Dose output was calibrated as 0.8 cGy/MU using identical reference conditions as the Halcyon linac.^33^

### 2.4 Planning techniques

Reference treatment plans were generated on the TrueBeam STx with HD 120 MLC (TB2‐SL2.5) and the TrueBeam ST with Millennium 120 MLC (TB2‐SL5) using a 6MV flattened dual arc VMAT planning technique according to our clinical practice and following the multicenter consensus of the FLAME consortium.^16^ The collimator angles were set at 10° and 80°. An avoidance sector between 170° and 190° gantry angle was used for both arcs to limit entrance dose in the rectum. The maximum dose rate for 6 MV flattened was used, namely 4.8 Gy/min in reference conditions. Manual treatment planning was performed for both TB2‐SL2.5 and TB2‐SL5.

**FIGURE 1:**
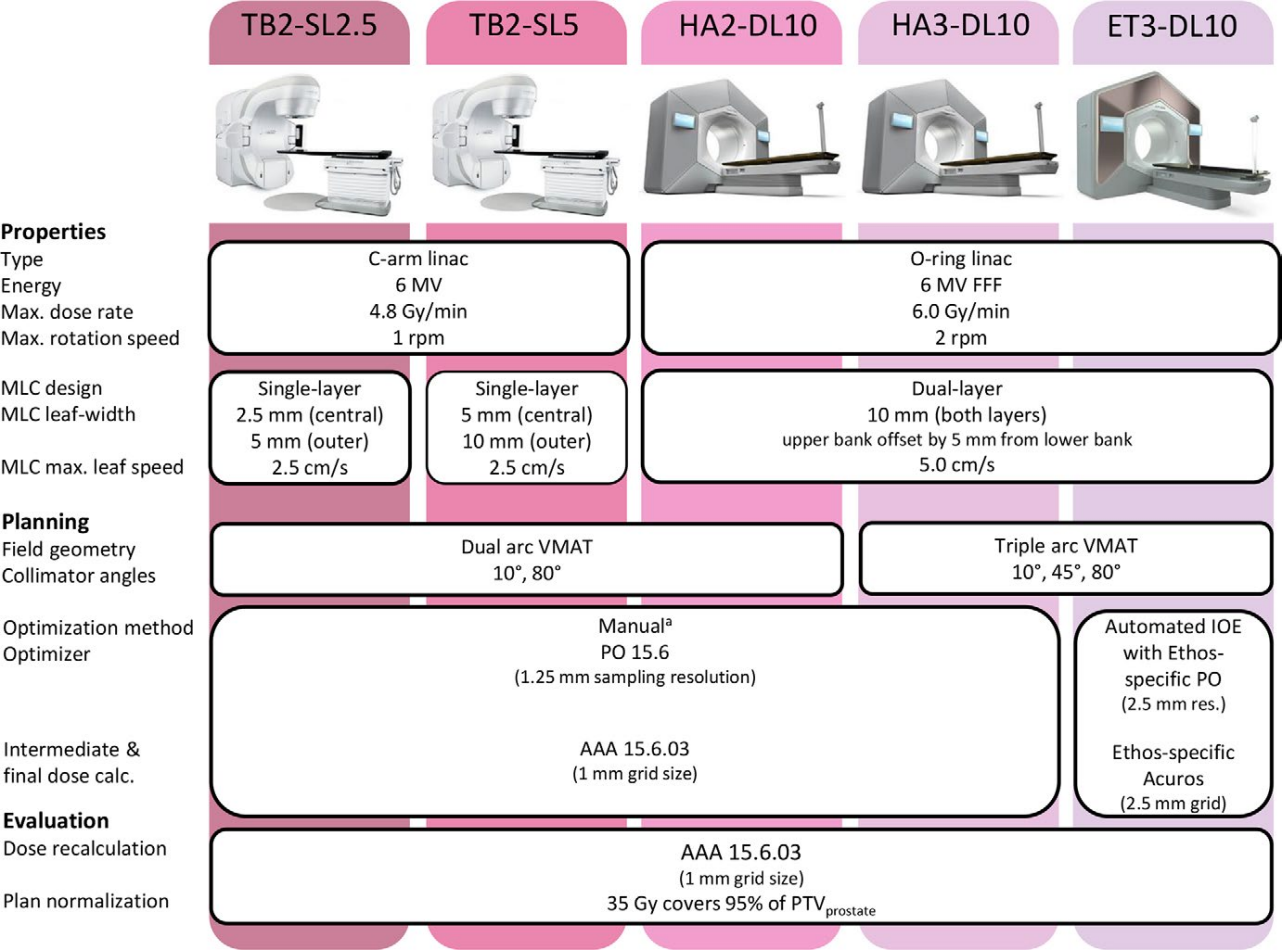
Schematic overview of the studied planning techniques. ^a^Manual treatment planning was first performed for TB2‐SL2.5 to determine the optimal patient‐specific set of optimization weights for the different objectives and the same patient‐specific set was used without modification to perform manual planning for TB2‐SL5, HA2‐DL10, and HA3‐DL10 (Figure 2).

The planning technique studied on the fast‐rotating O‐ring linac consisted of 6MV FFF dual arc VMAT using the same field geometry as on the C‐arm linacs, that is, collimator angles set at 10° and 80° and a rectal avoidance sector between 170° and 190° gantry angle. Given the maximum gantry rotation speed of 2 rpm during VMAT, it is hypothesized that an additional arc could be added to the planning technique without causing a major increase in treatment time. For this purpose, a 6 MV FFF triple arc VMAT solution was studied as well. The collimator angles were 10°, 45°, and 80° and the 45° angle was added to give more degrees of freedom to the optimizer for potentially improved OAR sparing.^34^ The maximum dose rate for 6 MV FFF was used, namely 6.0 Gy/min in reference conditions. Manual treatment planning was performed for both dual arc VMAT (HA2‐DL10) and triple arc VMAT (HA3‐DL10) on the fast‐rotating O‐ring linac. Automated treatment planning was performed solely for triple arc VMAT (ET3‐DL10) as it was not feasible to generate dual arc VMAT plans as the amount of MU that needed to be delivered per arc exceeded the machine tolerance limit (i.e., more than 1500 MU delivered per arc). This was due to the fact that it was not possible to constrain the amount of MU during automated plan optimization. In contrast, a maximum MU objective could be added for this purpose during manual treatment planning. An overview of the studied planning techniques is depicted in Figure 1.

### 2.5 Manual treatment planning

Manual treatment planning was performed in the Eclipse version 15.6 TPS using the Photon Optimizer (PO) algorithm version 15.6 with fine structure sampling resolution (1.25 mm grid size). The SX2 implementation of the Halcyon's dual‐layer MLC was used for HA2‐DL10 and HA3‐DL10 as this allowed for the field shaping to be performed by both MLC layers independently.

**FIGURE 2:**
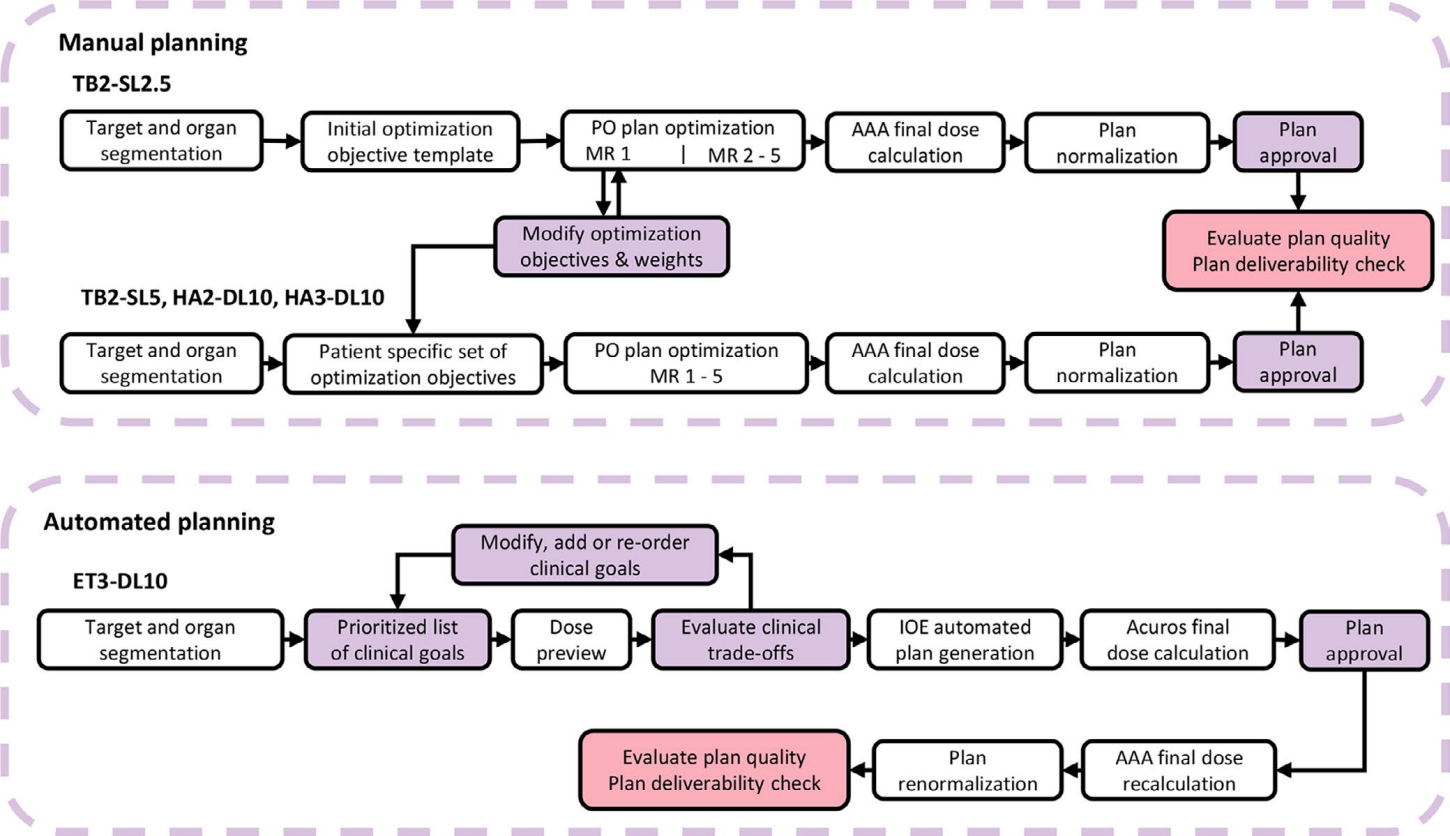
Schematic overview of the manual and automated treatment planning workflow. After automated planning, approved treatment plans were exported from Ethos Treatment Planning to Eclipse for final dose recalculation using the AAA version 15.6.03 algorithm with 1 mm calculation grid size and subsequent plan renormalization. The recalculated and renormalized plans were used for plan quality evaluation and plan deliverability verification.

Patient‐specific sets of optimization weights for the different objectives were determined for TB2‐SL2.5, which was the clinical planning technique used for the 16 patients treated on the hypo‐FLAME trial. The same patient‐specific set of optimization objectives and weights was used to generate the treatment plans for TB2‐SL5, HA2‐DL10, and HA3‐DL10 to minimize optimization‐based bias between the different manually optimized planning techniques. The patient‐specific optimization objectives and weights for TB2‐SL2.5 were determined through an iterative procedure during the first step of the first multiresolution level (MR 1) (Figure 2). The initial optimization objectives were obtained from an objective template (Supplemental Table S3) and were chosen to deliver a focal boost dose (D_99%_) to 40 Gy on GTV_boost_. The boost dose objective was then iteratively increased in steps of 1 up to 50 Gy. Sufficient time was left between subsequent iterations to allow for the cost function to flatten. All OAR dose constraints were verified and needed to be satisfied before further increasing the boost dose objective. The boost dose objective was reduced by 0.5 Gy if one of the OAR dose constraints was exceeded. The optimization was allowed to continue to higher multiresolution levels (MR 2–5) once the boost dose objective that resulted in the highest achievable focal boost dose was determined. In the case of multiple GTVs the optimal boost dose objective was determined for each GTV individually during the same optimization. During the remainder of the optimization, the other objectives in the objective template were to have a balanced competition between target coverage and the OAR objectives, especially the high‐dose spillage to the rectum and urethra. Once a clinically acceptable treatment plan was generated for TB2‐SL2.5, treatment plans for TB2‐SL5, HA2‐DL10, and HA3‐DL10 were generated using identical optimization objectives without any modifications during the optimization process.

A maximum monitor unit (MU) objective set to 2800 MU was used for TB2‐SL2.5, TB2‐SL5, and HA2‐DL10 in order for the treatment plans to be deliverable (i.e., to have less than 1500 MU delivered per arc). Such an objective was not used for HA3‐DL10 due to the additional arc and subsequent redistribution of the monitor units. The Automatic Optimization Mode and Automatic Intermediate Dose options were enabled to minimize manual interventions in the optimization process. Intermediate and final dose calculation was performed using the anisotropic analytical algorithm (AAA) version 15.6.03 with a 1 mm calculation grid size.

### 2.6 Automated treatment planning

Automated treatment planning was performed in the Physicians Intent module of Ethos Treatment Planning version 1.0 (Varian Medical Systems) using the Intelligent Optimization Engine (IOE). Clinical goals were defined for the targets and the OARs using a planning directive template and their respective priorities were specified on a scale from 1 (most important) to 4 (less important) (Supplemental Table S4). These clinical goals are converted by the IOE to optimization objectives for an Ethos‐specific variant of the Photon Optimizer algorithm, which performs the actual optimization. The IOE monitors this optimization process through a plan quality metric that is derived from the clinical goals and their relative priorities. During optimization, the IOE modifies the optimization objectives in order to maximize the plan quality metric while prioritizing failures in higher‐priority clinical goals before lower‐priority clinical goals.

Prior to automated planning, a fast fluence‐map optimized dose distribution was generated for the initial prioritized list of clinical goals using the IOE with the Fourier Transform Dose Calculation algorithm (FTDC) for dose calculation. This dose preview was used to evaluate potential clinical trade‐offs and the clinical goals were subsequently modified, added or re‐ordered to change their relative priority until a preview dose distribution was obtained that satisfied all the criteria of priority level 1 with only minor exceptions being allowed to the discretion of the planner (Figure 2). A strategy similar to the one performed during manual plan optimization was used to determine the optimal focal boost dose goal, that is, iterative escalation and de‐escalation of the GTV_boost_ D_99%_ clinical goal. Once the optimal clinical goals and their priorities were determined, the radiotherapy intent was approved and the IOE commenced with the automated planning.

The Ethos‐specific variant of the PO algorithm used a 2.5 mm structure sampling resolution. Intermediate and final dose calculation was performed using an Ethos‐specific variant of the Acuros XB algorithm with 2.5 mm calculation grid size using dose‐to‐medium. After automated planning, the generated plan was evaluated in Ethos Treatment Planning to assess whether it satisfied the planning objectives or whether the plan should be regenerated using a modified prioritized list of clinical goals. If the planning objectives were satisfied, the treatment plan was then exported to Eclipse for comparison.

### 2.7 Final dose recalculation and normalization for plan comparison

The dose distribution of the automatically generated treatment plans (ET3‐DL10) in Ethos Treatment Planning was recalculated in Eclipse version 15.6 using the AAA version 15.6.03 algorithm with a 1 mm calculation grid size to minimize calculation‐based bias in the comparison with the manually generated treatment plans in Eclipse (Figure 2). The dose distribution of all plans was normalized such that the prescribed dose of 35 Gy covers 95% of PTV_prostate_. These normalized dose distributions were used for the plan quality evaluation.

### 2.8 Plan quality evaluation

Target coverage and OAR dose metrics specified in Table 2 were evaluated for all plans. Normal tissue complication probability (NTCP) for the rectum and the bladder were calculated using the Lyman‐Kutcher‐Burman model with Niemierko's equivalent uniform dose as described in the Supplemental Material.^35^, ^36^, ^37^ Rectum NTCP was calculated using the best estimate QUANTEC parameters for late grade ≥2 rectal toxicity or bleeding.^38^ Bladder NTCP was calculated using the parameters derived by Kole et al. for late urinary symptom flare after prostate SBRT in five fractions.^39^ The rectum DVH was corrected for the biologically effective dose using an α/β ratio of 3 Gy before calculating the rectum NTCP.^38^, ^40^ The original bladder DVH could be used for the bladder NTCP calculation as the fractionation scheme of this study is identical to that of the model.

The target coverage, OAR dose metrics, and NTCP values obtained by the different planning techniques were compared using a Kruskal–Wallis omnibus test followed by two‐sided Wilcoxon's matched‐pairs signed‐rank tests. Statistical significance was determined using the Benjamini‐Hochberg procedure to correct for multiple comparisons by controlling the false discovery rate at significance level 0.05.^41^ All analyses were performed in MATLAB R2017b (MathWorks, Natick, MA, USA).

### 2.9 Plan deliverability

The deliverability of all treatment plans was verified through pre‐treatment patient‐specific quality assurance (QA) using portal image dosimetry. The agreement between measured and calculated planar measurements was analyzed for each arc individually using a 2%(local)/2 mm gamma index (γ_2%,2mm_) analysis with an exclusion threshold set to 20% of the maximum value.^42^ The gamma index analysis was performed in Portal Dosimetry (Varian Medical Systems). Treatment plans passed the clinical tolerance limit and were deemed deliverable if the γ_2%,2mm_ agreement score was ≥95% for each arc individually as recommended by the AAPM TG‐218 report.^43^ The time needed to deliver the dose was recorded for all plans during treatment plan verification using the auto‐sequencing delivery mode.

## 3 RESULTS

### 3.1 Target coverage and high and intermediate dose spillage

**FIGURE 3:**
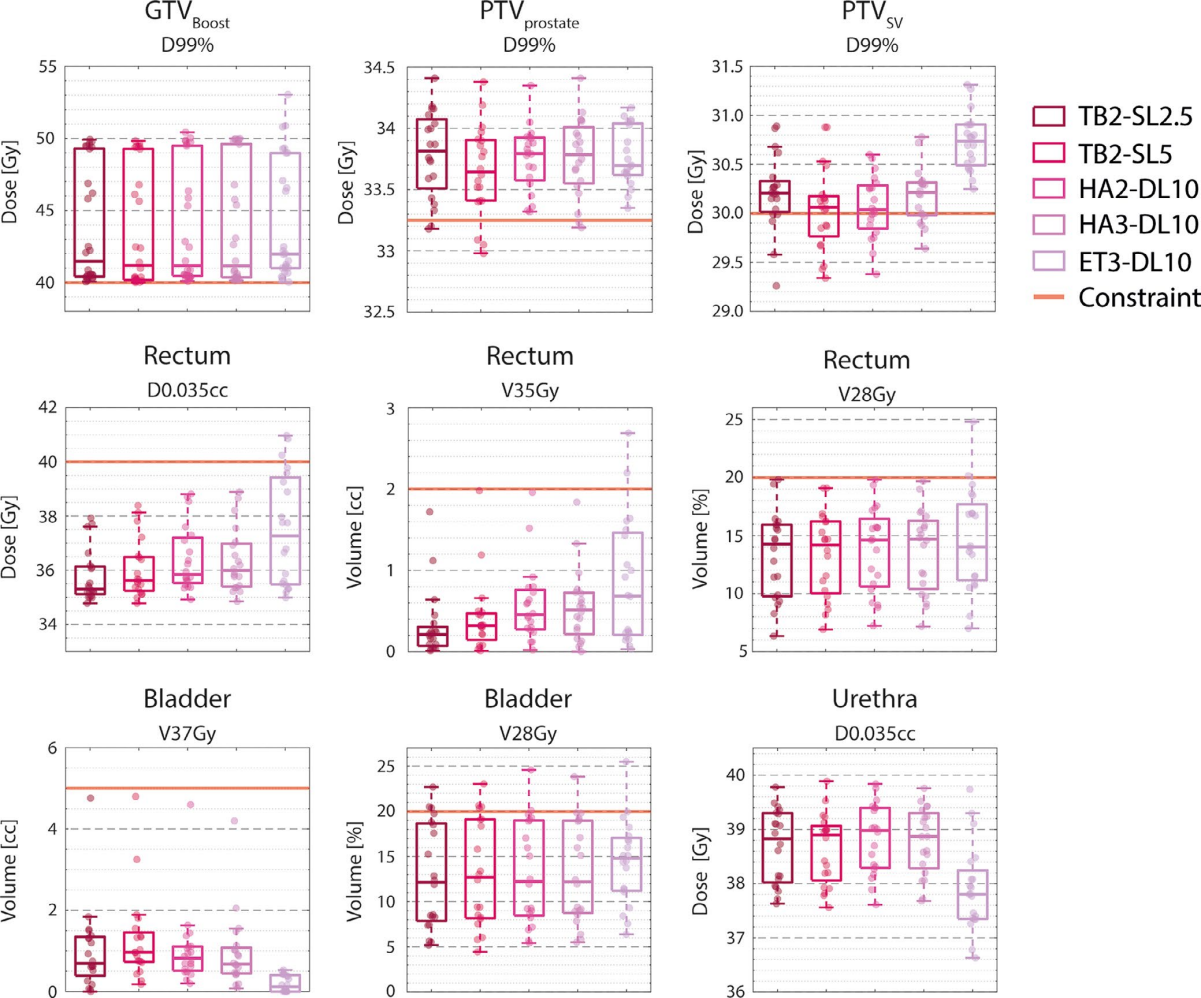
Target coverage and dose to selected organs at risks. Results are shown as boxplots over all patients for TB2‐SL2.5 (outer left), TB2‐SL5 (middle left), HA2‐DL10 (middle), HA3‐DL10 (middle right) and ET3‐DL10 (outer right). The dots represent individual patients. Target volume dose prescriptions and OAR dose constraints are indicated by a red solid line. The corresponding values are presented in Tables 3 and 4.

The PTV_prostate_ was adequately covered in all patients for all plans whereas the PTV_SV_ had a minor deviation from its dose prescription in 24 of the 100 treatment plans (mostly for TB2‐SL5 with a violation in 9/20 plans). A minimal focal boost to D_99%_ ≥ 40.0 Gy on GTV_boost_ was achieved for all treatment plans. The D_99%_ to PTV_prostate_ was similar for all planning techniques (Figure 3). The D_99%_ to PTV_SV_ was statistically significant (*p* < 0.001) and consistently higher for ET3‐DL10 compared to all other planning techniques (median increase ranging between 0.5 and 0.7 Gy). The D_99%_ to GTV_boost_ was slightly higher for ET3‐10mm compared to the other planning techniques (median increase ranging between 0.2 Gy and 0.3 Gy) but this increase was not statistically significant nor consistent for all patients. Comparable high dose spillage (CI) was observed for all planning techniques (Supplemental Figure 1). In contrast, ET3‐DL10 had a statistically significant (*p* < 0.001) and consistently higher intermediate dose spillage measured by D2cm compared to all other planning techniques (median increase ranging from 7.1% to 9.8%). A statistically significant (*p* < 0.001) albeit smaller increase in D2cm was found for TB2‐SL5 (median 1.2%), HA2‐DL10 (2.5%), and HA3‐DL10 (1.4%) compared to TB2‐SL2.5.

More details on the target coverage and high and intermediate dose spillage are presented in Table 3 and are visualized in Supplemental Figure S1. The pairwise dosimetric comparison and the corresponding *p*‐values of the statistical analysis are presented in Supplemental Tables S4 and S5.

**TABLE 3:** Evaluation of target coverage and high and intermediate dose spillage

	Constraint	TB2‐2.5 mm	TB2‐5 mm	HA2‐10 mm	HA3‐10 mm	ET3‐10 mm
		Median (Range)	Median (Range)	Median (Range)	Median (Range)	Median (Range)
Target coverage						
GTV_boost_						
D_99%_ [Gy]	>40	41.5 (40.1, 49.9)	41.2 (40.0, 49.8)	41.2 (40.1, 50.4)	41.2 (40.1, 50.0)	42.0 (40.0, 53.0)
D_0.1cc_ [Gy]	<52	47.8 (42.0, 51.4)	47.4 (42.5, 51.7)	47.0 (42.8, 52.5)	47.2 (43.0, 52.5)	45.1 (42.0, 55.3)
PTV_prostate_						
D_99%_ [Gy]	>33.25	33.8 (33.2, 34.4)	33.7 (33.0, 34.4)	33.8 (33.3, 34.4)	33.8 (33.2, 34.4)	33.7 (33.4, 34.2)
PTV_SV_						
D_99%_ [Gy]	>30	30.2 (29.3, 30.9)	30.1 (29.3, 30.9)	30.0 (29.4, 30.6)	30.2 (29.6, 30.8)	30.7 (30.3, 31.3)
Dose spillage						
CI		1.13 (1.08, 1.24)	1.15 (1.08, 1.24)	1.16 (1.08, 1.27)	1.14 (1.08, 1.23)	1.18 (1.09, 1.43)
D2cm [%]		53.8 (51.7, 56.7)	54.8 (52.6, 58.2)	56.3 (52.4, 59.8)	55.4 (52.7, 58.2)	63.5 (61.3, 69.6)

Note

Median and range (min, max) over all 20 patients of each planning technique are given for all parameters. The conformity index (CI) and dose at 2 cm (D2cm) are calculated for PTV_prostate_ and a prescribed dose of 35 Gy.

### 3.2 Dose to OAR

The maximum dose D_0.035cc_ ≤ 40 Gy constraint to the rectum was exceeded in three treatment plans (all ET3‐DL10). Similarly, the rectum V_35 Gy_ exceeded 2.0 cc in two ET3‐DL10 plans (V_35 Gy_ of 2.20 and 2.69 cc). However, the second patient case for which the constraint was violated had a large overlap between the rectum and the PTV_prostate_ (overlap of 3.71 cc) and all other planning techniques also just met the dose constraint (V_35 Gy_ between 1.72 cc and 1.98 cc) (Figure 3). Although the rectum V_38 Gy_ dose constraint was satisfied for all patients, a statistically significant (*p* < 0.001) and the consistent increase was observed for ET3‐DL10 compared to all other planning techniques (median increase of 0.02 cc). Comparable intermediate dose to the rectum (i.e., V_32 Gy_, V_28 Gy_, V_23.5 Gy_, and V_17 Gy_) was observed for all planning techniques (Supplemental Figure S2). The rectum NTCP for late grade ≥2 rectal complications showed a slight increase for ET3‐DL10 compared to the other techniques (median increase ranging between 0.4% and 0.5%) but this difference was not statistically significant nor consistent for all patients (Supplemental Figure S2).

The bladder V_32 Gy_ ≤ 15% and V_28 Gy_ ≤ 20% constraints were exceeded in one patient by all planning techniques (V_32 Gy_ between 15.5% and 16.1% and V_28 Gy_ between 22.7% and 25.5%), which may be related to 13.2% of the bladder volume overlapping with the PTV_prostate_. ET3‐DL10 had a statistically significant (*p* < 0.001) and consistent reduction in V_37 Gy_ to the bladder (median decrease ranging between −0.69 cc and −0.57 cc). Comparable intermediate dose to the bladder (i.e., V_32 Gy_ and V_38 Gy_) was observed for all planning techniques. ET3‐DL10 showed a slight reduction in bladder NTCP for late urinary symptom flare (Supplemental Figure S3) but no statistically significant difference in NTCP was found between the different planning techniques.

The maximum dose of D_0.035cc_ to the urethra was below 39.9 Gy for all treatment plans (Figure 3). A statistically significant (*p* < 0.001) and consistent reduction in urethra D_0.035cc_ was observed for ET3‐DL10 compared to all other planning techniques (median decrease ranging between −1.1 and −0.9 Gy).

All plans satisfied the dose constraints to the anal canal and penile bulb (Supplemental Figure S4). There were no statistically significant differences between the different planning techniques.

More details on the dose to the OAR and NTCP are presented in Table 4 and are visualized in Supplemental Figures S2‐S5. The pairwise dosimetric comparison and the corresponding *p*‐values of the statistical analysis are presented in Supplemental Table S4 and S5.

**TABLE 4:** Evaluation of dose to organs at risk (OAR). and normal tissue complication probability (NTCP)

	Constraint	TB2‐2.5mm	TB2‐5mm	HA2‐10mm	HA3‐10mm	ET3‐10mm
		Median (Range)	Median (Range)	Median (Range)	Median (Range)	Median (Range)
Dose to OAR						
Rectum						
D_0.035cc_ [Gy]	≤40	35.3 (34.8, 37.9)	35.6 (34.8, 38.4)	35.9 (34.9, 38.8)	36.0 (34.9, 38.9)	37.3 (35.0, 41.0)
V_38 Gy_ [cc]	≤1	0.00 (0.00, 0.03)	0.00 (0.00, 0.06)	0.00 (0.00, 0.11)	0.00 (0.00, 0.10)	0.02 (0.00, 0.69)
V_35 Gy_ [cc]	≤2	0.21 (0.01, 1.72)	0.32 (0.01, 1.98)	0.46 (0.02, 1.96)	0.52 (0.00, 1.84)	0.69 (0.00, 2.69)
V_32 Gy_ [%]	≤15	7.9 (2.6, 13.8)	8.0 (2.5, 12.7)	7.8 (2.5, 13.2)	7.7 (2.5, 12.9)	8.5 (2.2, 17.5)
V_28 Gy_ [%]	≤20	14.3 (6.3, 19.8)	14.2 (6.9, 19.1)	14.6 (7.2, 19.8)	14.7 (7.2, 19.7)	14.0 (7.0, 24.8)
V_23.5 Gy_ [%]	≤50	20.2 (12.9, 25.3)	20.3 (13.4, 27.0)	21.0 (14.0, 26.7)	21.4 (14.0, 26.1)	21.1 (12.7, 30.7)
V_20.5 Gy_ [%]	≤70	24.5 (17.9, 31.5)	24.6 (17.3, 31.0)	25.0 (17.5, 30.7)	25.3 (17.5, 30.0)	25.0 (16.6, 34.6)
V_17 Gy_ [%]	≤75	28.8 (23.8, 34.5)	29.5 (22.7, 34.4)	29.8 (22.5, 35.3)	29.9 (22.3, 33.7)	29.9 (22.4, 40.0)
PRV_rectum_						
D_0.035cc_ [Gy]	≤42	39.9 (36.5, 41.8)	40.0 (36.3, 41.7)	40.1 (36.5, 42.6)	40.1 (36.4, 42.8)	40.4 (36.5, 42.2)
Bladder						
V_42 Gy_ [cc]	≤1	0.00 (0.00, 0.03)	0.00 (0.00, 0.04)	0.00 (0.00, 0.15)	0.00 (0.00, 0.07)	0.00 (0.00, 0.00)
V_37 Gy_ [cc]	≤5	0.70 (0.00, 4.76)	0.97 (0.18, 4.80)	0.82 (0.20, 4.60)	0.68 (0.08, 4.20)	0.12 (0.00, 0.53)
V_32 Gy_ [%]	≤15	7.2 (2.9, 15.8)	7.5 (3.1, 15.8)	6.9 (2.9, 15.9)	6.9 (2.8, 15.5)	7.6 (3.3, 16.1)
V_28 Gy_ [%]	≤20	12.1 (5.2, 22.7)	12.7 (4.5, 23.0)	12.2 (5.4, 24.6)	12.2 (5.5, 23.8)	14.8 (6.4, 25.5)
Urethra						
D_0.035cc_ [Gy]	≤42	38.8 (37.6, 39.8)	38.9 (37.6, 39.9)	39.0 (37.6, 39.8)	38.9 (37.7, 39.8)	37.8 (36.6, 39.7)
PRV_urethra_						
D_0.035cc_ [Gy]	≤42	39.7 (38.2, 41.1)	39.8 (38.4, 41.3)	40.0 (38.6, 41.2)	40.0 (38.6, 41.1)	40.4 (37.8, 42.0)
Anal canal						
V_37.85 Gy_ [cc]	≤1	0.00 (0.00, 0.00)	0.00 (0.00, 0.00)	0.00 (0.00, 0.00)	0.00 (0.00, 0.00)	0.00 (0.00, 0.00)
V_21.4 Gy_ [%]	≤40	2.7 (0.0, 37.3)	2.7 (0.0, 37.2)	3.2 (0.0, 35.7)	3.6 (0.0, 37.7)	2.5 (0.0, 37.2)
V_19.2 Gy_ [%]	≤60	3.7 (0.0, 38.9)	3.6 (0.0, 39.5)	4.2 (0.0, 37.6)	4.5 (0.0, 39.4)	3.2 (0.0, 40.9)
Penile bulb						
V_20 Gy_ [%]	≤90	0.0 (0.0, 24.9)	0.0 (0.0, 42.7)	0.0 (0.0, 64.2)	0.0 (0.0, 48.2)	0.0 (0.0, 42.2)
Femoral head & neck						
V_28 Gy_ [%]	≤5	0.0 (0.0, 0.0)	0.0 (0.0, 0.0)	0.0 (0.0, 0.0)	0.0 (0.0, 0.0)	0.0 (0.0, 0.0)
**NTCP**						
Rectum^a^ [%]		1.0 (0.2, 2.7)	1.0 (0.2, 2.9)	1.1 (0.2, 3.4)	1.2 (0.2, 3.2)	1.2 (0.2, 8.1)
Bladder^b^ [%]		4.2 (1.9, 10.6)	4.4 (1.9, 10.8)	4.2 (1.9, 11.0)	4.2 (1.8, 10.6)	4.3 (1.9, 8.8)

Note

Median and range (min, max) over all 20 patients of each planning technique are given for all parameters.

^a^ Grade ≥2 late toxicity or rectal bleeding.^38^

^b^ Late urinary flare.^39^

### 3.3 Beam delivery parameters and pre‐treatment patient‐specific QA

All plans passed the patient‐specific QA tolerance limit with γ_2%/2mm_ agreement scores (AS) above 95.0% and were deemed deliverable. In general, the dose delivery time of HA2‐DL10 (median of 3 min 52 s) and HA3‐DL10 (4 min 6 s) was shorter than those of TB2‐SL2.5 (4 min 29 s) and TB2‐SL5 (4 min 27 s). In contrast, ET3‐DL10 had the longest dose delivery time of all the planning techniques with a median of 5 min 2 s. More details on the beam delivery parameters and pre‐treatment patient‐specific QA are given in Table 5. The monitor units and beam‐on time are visualized in Supplemental Figure S6.

**TABLE 5:** Beam delivery parameters and pre‐treatment patient‐specific quality assurance (QA)

	TB2‐2.5mm	TB2‐5mm	HA2‐10mm	HA3‐10mm	ET3‐10mm
	Median (Range)	Median (Range)	Median (Range)	Median (Range)	Median (Range)
Beam delivery parameters					
Monitor Units [MU]^a^	2605 (2407, 2844)	2614 (2341, 2809)	2255 (1938, 2798)	2382 (2088, 2874)	2890 (2371, 3380)
MU per arc [MU]^a^	1314 (1130, 1499)	1303 (1121, 1484)	1111 (952, 1445)	790 (628, 1107)	962 (684, 1362)
Beam‐on time [min:sec]	4:29 (4:07, 4:52)	4:27 (4:01, 4:48)	3:52 (3:23, 4:51)	4:06 (3:24, 4:59)	5:02 (3:48, 5:49)
Beam‐on time per arc [min:sec]	2:13 (1:55, 2:32)	2:12 (1:54, 2:30)	1:54 (1:38, 2:27)	1:18 (1:03, 1:54)	1:40 (1:11, 2:21)
γ_2%/2mm_ agreement score [%]	99.2 (97.3, 100.0)	99.7 (97.8, 100.0)	99.7 (96.4, 100.0)	99.0 (95.0, 100.0)	99.8 (98.6, 100.0)

Note

Median and range (min, max) over all 20 plans of each planning technique are given for all parameters.

^a^ Different absolute dose calibrations were used for the TrueBeam linacs and the Halcyon: 1 MU corresponds to 0.8 cGy for TrueBeam and 1.0 cGy for Halcyon, both for a 10 × 10 cm² field at 10 cm depth with an SSD of 90 cm.

## 4 DISCUSSION

This study investigated the feasibility of automated treatment planning of prostate SBRT with focal boosting on the Halcyon fast‐rotating O‐ring linac as a first step in the implementation of online adaptive radiotherapy of this treatment on the Ethos therapy platform. For this purpose, automated treatment planning was performed for VMAT on the fast‐rotating O‐ring linac (ET3‐DL10) and was compared to the manual treatment planning of VMAT on standard clinical C‐arm linacs (TB2‐SL2.5 and TB2‐SL5). This allowed to investigate whether similar plan quality could be achieved on the system as those obtained by the planning techniques that were used to deliver the treatment on the hypo‐FLAME trial.^16^ It should be noted that the plan quality obtained by ET3‐DL10 is determined by the combination of the performance of the dual‐layer MLC system and the automated planning algorithm of Ethos Treatment Planning. By also performing manual treatment planning on the fast‐rotating O‐ring linac (HA2‐DL10 and HA3‐DL10) it was possible to separate the influence of both factors from each other and evaluate them individually.

To minimize optimization‐based bias during manual treatment planning, identical patient‐specific sets of optimization objectives were used for TB2‐SL2.5, TB2‐SL5, HA2‐DL10, and HA3‐DL10. In this way, the performance of each MLC system on the plan quality could be evaluated without it being distorted by differences in the optimization process. The optimization objectives and weights were determined for each patient during the plan optimization of TB2‐SL2.5 following a predetermined iterative procedure as described in the methods (Figure 2). Since TB2‐SL2.5 was the clinical planning technique for the 16 patients treated on the hypo‐FLAME trial its plan quality should be representative of the plan quality obtained in clinical practice.^16^ A potential limitation of this approach, however, is that the obtainable focal boost dose and OAR sparing of TB2‐SL5, HA2‐DL10, and HA3‐DL10 will be limited by those achieved on TB2‐SL2.5 as its optimization objectives and weights were used to drive the optimization of the other planning techniques.

It should be noted, that automated treatment planning uses an Ethos‐specific variant of the Acuros XB algorithm for the intermediate dose calculation in the Ethos Treatment Planning software whereas the plan quality evaluation and plans deliverability verification was performed using the recalculated and renormalized plans in Eclipse (Figure 2). As a consequence, the evaluated dose distribution of ET3‐DL10 in Eclipse may differ from the optimized dose distribution in Ethos Treatment Planning. However, this influence on the plan quality comparison was limited as the median voxel‐wise dose error between both dose distributions was below 0.2 Gy for the dose points above 20% of the prescribed dose of 35 Gy. In addition, the dose rescaling factor needed to renormalize the recalculated dose distribution was below 2% for all plans.

All planning techniques on the fast‐rotating O‐ring linac were capable to generate clinically acceptable treatment plans (Tables 3 and 4). When comparing triple arc VMAT (HA3‐DL10) to dual arc VMAT (HA2‐DL10), no clinically significant differences were found in terms of target coverage or OAR doses, indicating that adding a third arc does not improve plan quality in general (Supplemental Tables S4 and S5). As hypothesized, adding a third arc redistributed the amount of MU (median of 790 MU/arc for HA3‐DL10 vs. 1111 MU/arc for HA2‐DL10) and did not cause a major increase in dose delivery time (4 min 6 s vs. 3 min 52 s). This MU redistribution over the three arcs was, however, necessary to generate deliverable plans through automated planning as there was no way to constrain the amount of MU in Ethos Treatment Planning. This caused the dual arc VMAT field geometry to exceed the machine tolerance limit (>1500 MU delivered per arc) such that automated planning was only performed for triple arc VMAT (ET3‐DL10). However, this issue may be resolved in an upcoming version of the Ethos Treatment Planning software.

Benchmarking ET3‐DL10 with the plan quality obtained by the C‐arm linacs (TB2‐SL2.5 and TB2‐SL5) revealed an increase in dose coverage (D_99%_) to PTV_SV_, an increase in intermediate dose spillage (D2cm), an increase in high‐dose spillage to the rectum (V_38 Gy_) and a reduction in high‐dose spillage to both the bladder (V_37 Gy_) and the urethra (D_0.035cc_) for ET3‐DL10. Most importantly, these statistically significant differences were not observed when benchmarking HA2‐DL10 and HA3‐DL10 with the C‐arm linacs as all these planning techniques demonstrated comparable plan quality (Figure 3). This latter observation is consistent with the results obtained by Pokhrel et al., which compared the plan quality of dual‐arc VMAT on the fast‐rotating O‐ring linac with dual‐arc VMAT on a conventional C‐arm linac for whole‐gland prostate SBRT and also found no statistically significant differences.^44^

As such, the observed differences between ET3‐DL10 and the other planning techniques are presumably the result of the difference in the actual optimization process between automated and manual plan optimization. The main difference between both optimization processes lies in the determination and adjustment of the optimization objectives during optimization. In manual treatment planning these were explicitly defined by the planner and were not manipulated throughout the optimization process once the patient‐specific set of optimization objectives was determined (Figure 2). In contrast, in automated treatment planning, the optimization objectives are only implicitly defined through a prioritized list of clinical goals and it is the IOE that determines the optimization objectives based on this list. Moreover, objective manipulations were also performed by the IOE during optimization to maximize the plan quality metric and these manipulations cannot be examined by the planner. Hence, there will undoubtedly be a difference in the optimization objectives used between both planning methods influencing the final plan. In addition, automated treatment planning uses a newer, specific version of the PO algorithm integrated into the IOE which differs from the PO version 15.6 algorithm used in Eclipse. For instance, a different multi‐resolution schedule is used in this Ethos‐specific variant compared to the standard Eclipse version. There was also a difference in the structure sampling resolution (2.5 vs. 1.25 mm) and the dose grid size (2.5 vs. 1 mm) of the intermediate dose calculation between automated and manual treatment planning.

It is important to note that despite certain increases in OAR dose, most OAR dose values were well below the dose constraints used in the hypo‐FLAME trial.^16^ This is reflected by the low NTCP values for late grade ≥2 rectal toxicity or bleeding (≤4.0% when neglecting the patient with large rectum/PTV_prostate_ overlap) and late urinary symptom flare (≤7.6% when neglecting the patient with large bladder/PTV_prostate_ overlap) that were estimated in this study. To our knowledge, there is currently no rectal NTCP model available whose parameters are derived from an ultra‐hypofractionated treatment schedule. For this reason, a well‐established NTCP model in the conventionally fractionated setting, the best estimate QUANTEC parameters,^38^ was used to estimate the incidence of rectal toxicity. To correct for the difference in fractionation schemes, a radiobiological effective dose correction was performed prior to NTCP calculation using an α/β ratio of 3 Gy as recommended by the QUANTEC study on rectal toxicity.^38^ This estimate is supported by the late rectal toxicity endpoint analysis of the CHHiP trial^40^ and by the results from the HYPO‐RT‐PC trial, which assumed a late rectal α/β of 3 Gy and showed isoeffective cumulative grade 2 or worse rectal toxicity in both arms.^12^ Calculated NTCP values in our study were in line with the long‐term outcomes of prospective clinical trials of prostate SBRT without focal boosting (weighted incidence of 4.9%).^10^, ^11^ To estimate the incidence of bladder toxicity, the parameters derived by Kole et al. for late urinary symptom flare after prostate SBRT in five fractions were used as this allows for a direct NTCP calculation without the need to correct for differences in fractionation schemes.^39^ Calculated NTCP values were similar to those previously reported in the literature (incidence of 13.4%).^45^ Most importantly, no statistically nor clinically significant differences were found when comparing the NTCP values of all planning techniques.

Plan deliverability was assessed through pre‐treatment patient‐specific QA using portal image dosimetry. All treatment plans were found to be within the tolerance limits of the AAPM TG‐218 report for IMRT measurement‐based verification QA as portal image dosimetry measurements had γ_2%/2mm_ agreement scores above 95% (Table 5).^43^ These patient‐ specific QA results are consistent with previous reports on the treatment delivery quality of VMAT on the fast‐rotating O‐ring linac.^44^, ^46^, ^47^

This work constitutes a first step in the implementation of online treatment plan adaptation for prostate SBRT with focal boosting using the Ethos therapy treatment platform. The impact of both the dual‐layer MLC’s performance and the platform's automated planning algorithms on the plan quality of this treatment were investigated separately. It was found that the dual‐layer MLC system with its 10 mm leaf width could achieve similar plan quality as those obtained by standard clinical C‐arm linacs with a single‐layer MLC system with smaller leaf width. Moreover, automated treatment planning was feasible and resulted in similar plan quality as those obtained through manual treatment planning. Hence, automated planning on the fast‐rotating O‐ring linac generated treatment plans that are of similar quality as those currently used in clinical practice. However, certain differences were present due to the inherent difference in which the optimization is performed between both planning approaches. Further fine‐tuning of the clinical goals may allow the optimization of the automated planning process and could reduce the observed differences in plan quality between both approaches. Nonetheless, the findings presented in this work now make it possible to further explore the potential benefits of online adaptive radiotherapy on the Ethos system for prostate SBRT with focal boosting.

The RATING guidelines for treatment planning studies^48^ were used to revise the manuscript. The corresponding author concluded that the RATING score was 90% (RATING score sheet included in Supplemental Material).

## 5 CONCLUSION

Automated treatment planning of prostate SBRT with focal boosting on the fast‐rotating O‐ring linac is feasible and achieves similar plan quality as those obtained for standard clinical C‐arm linacs using manual treatment planning. The observed differences in plan quality are mainly caused by the difference in translating the planning objectives into optimization objectives (manual planning) or clinical goals (automated planning) used to drive optimization, while the performance of the dual‐layer MLC has only a moderate impact on the plan quality. Adding a third arc does not improve plan quality in general but is currently necessary to generate clinically deliverable treatment plans with automated planning as dual arc VMAT plans generated this way exceeds the machine tolerance limit for the amount of MU that can be delivered per arc and are thus not deliverable.

## CONFLICTS OF INTEREST

This work was performed within a collaboration agreement between Varian Medical Systems and the Department of Radiation Oncology at the University Hospitals Leuven.

## AUTHOR CONTRIBUTION

RDR, TD, and WC conceived the project and study methodology. RDR collected data, performed measurements, and analyzed the data. CD, KP, and BDW provided clinical expertise. RDR wrote the manuscript and all co‐authors reviewed, revised, and approved the final manuscript. TD supervised the project.

## Supporting information

Supplementary Material

Supplementary Material

## Data Availability

The data that support the findings of this study are available from the corresponding author upon reasonable request.

## References

[1] Hamdy FC, Donovan JL, Lane JA, et al. 10‐year outcomes after monitoring, surgery, or radiotherapy for localized prostate cancer. N Engl J Med. 2016;375(15):1415‐1424. 27626136 10.1056/NEJMoa1606220

[2] Dearnaley DP, Jovic G, Syndikus I, et al. Escalated‐dose versus control‐dose conformal radiotherapy for prostate cancer: long‐term results from the MRC RT01 randomised controlled trial. Lancet Oncol. 2014;15(4):464‐473. 24581940 10.1016/S1470-2045(14)70040-3

[3] Kupelian PA, Ciezki J, Reddy CA, Klein EA, Mahadevan A. Effect of increasing radiation doses on local and distant failures in patients with localized prostate cancer. Int J Radiat Oncol Biol Phys. 2008;71(1):16‐22. 17996382 10.1016/j.ijrobp.2007.09.020

[4] Pahlajani N, Ruth KJ, Buyyounouski MK, et al. Radiotherapy doses of 80 Gy and higher are associated with lower mortality in men with Gleason score 8 to 10 prostate cancer. Int J Radiat Oncol Biol Phys. 2012;82(5):1949‐1956. 21763081 10.1016/j.ijrobp.2011.04.005 PMC3827957

[5] Cellini N, Morganti AG, Mattiucci GC, et al. Analysis of intraprostatic failures in patients treated with hormonal therapy and radiotherapy: implications for conformal therapy planning. Int J Radiat Oncol Biol Phys. 2002;53(3):595‐599. 12062602 10.1016/s0360-3016(02)02795-5

[6] Monninkhof EM, van Loon JWL, van Vulpen M, et al. Standard whole prostate gland radiotherapy with and without lesion boost in prostate cancer: toxicity in the FLAME randomized controlled trial. Radiother Oncol. 2018;127(1):74‐80. 29336835 10.1016/j.radonc.2017.12.022

[7] Murray JR, Tree AC, Alexander E, et al. Standard and hypofractionated dose escalation to intraprostatic tumour nodules in localised prostate cancer: efficacy and toxicity in the DELINEATE trial. Int J Radiat Oncol. 2019;106(4):715‐724. 10.1016/j.ijrobp.2019.11.402 31812718

[8] Kerkmeijer LGW, Groen VH, Pos FJ, Haustermans K. Focal boost to the intraprostatic tumor in external beam radiotherapy for patients with localized prostate cancer: results from the FLAME randomized phase III trial. J Clin Oncol. 2021;1‐11. 33471548 10.1200/JCO.20.02873

[9] Draulans C, van der Heide UA, Haustermans K, et al. Primary endpoint analysis of the multicentre phase II hypo‐FLAME trial for intermediate and high risk prostate cancer. Radiother Oncol. 2020;147:92‐98. 32247206 10.1016/j.radonc.2020.03.015

[10] Kishan AU, Dang A, Katz AJ, et al. Long‐term outcomes of stereotactic body radiotherapy for low‐risk and intermediate‐risk prostate cancer. JAMA Netw Open. 2019;2(2):1‐13. 10.1001/jamanetworkopen.2018.8006 PMC6484596 30735235

[11] Jackson WC, Silva J, Hartman HE, et al. Stereotactic body radiotherapy for localized prostate cancer: a systematic review and meta‐analysis of over 6,000 patients treated on prospective studies. Int J Radiat Oncol. 2019;104(4):778‐789. 10.1016/j.ijrobp.2019.03.051 PMC6770993 30959121

[12] Widmark A, Gunnlaugsson A, Beckman L, et al. Ultra‐hypofractionated versus conventionally fractionated radiotherapy for prostate cancer: 5‐year outcomes of the HYPO‐RT‐PC randomised, non‐inferiority, phase 3 trial. Lancet. 2019;6736(19):1‐11. 10.1016/S0140-6736(19)31131-6 31227373

[13] Brand DH, Tree AC, Ostler P, et al. Intensity‐modulated fractionated radiotherapy versus stereotactic body radiotherapy for prostate cancer (PACE‐B): acute toxicity findings from an international, randomised, open‐label, phase 3, non‐inferiority trial. Lancet Oncol. 2019;2045(19):1‐13. 10.1016/S1470-2045(19)30569-8 PMC6838670 31540791

[14] Zietman AL. Making radiation therapy for prostate cancer more economical and more convenient. J Clin Oncol. 2016;34(20):2323‐2324. 27091711 10.1200/JCO.2016.67.3764

[15] Ghadjar P, Fiorino C, Munck af Rosenschöld P, Pinkawa M, Zilli T, van der Heide UA. ESTRO ACROP consensus guideline on the use of image guided radiation therapy for localized prostate cancer. Radiother Oncol. 2019;141:5‐13. 31668515 10.1016/j.radonc.2019.08.027

[16] Draulans C, De Roover R, van der Heide UA, et al. Stereotactic body radiation therapy with optional focal lesion ablative microboost in prostate cancer: topical review and multicenter consensus. Radiother Oncol. 2019;140:131‐142. 31276989 10.1016/j.radonc.2019.06.023

[17] Deutschmann H, Kametriser G, Steininger P, et al. First clinical release of an online, adaptive, aperture‐based image‐guided radiotherapy strategy in intensity‐modulated radiotherapy to correct for inter‐ and intrafractional rotations of the prostate. Int J Radiat Oncol Biol Phys. 2012;83(5):1624‐1632. 22209149 10.1016/j.ijrobp.2011.10.009

[18] Njeh CF, Snyder KC, Cai J. The use of six degrees of freedom couch is only clinically beneficial in stereotactic radio surgery. Med Phys. 2019;46(2):415‐418. 30620084 10.1002/mp.13380

[19] Lips IM, van der Heide UA, Kotte ANTJ, van Vulpen M, Bel A. Effect of translational and rotational errors on complex dose distributions with off‐line and on‐line position verification. Int J Radiat Oncol Biol Phys. 2009;74(5):1600‐1608. 19473778 10.1016/j.ijrobp.2009.02.056

[20] Shang Q, Olsen LJS, Stephans K, Tendulkar R, Xia P. Prostate rotation detected from implanted markers can affect dose coverage and cannot be simply dismissed. J Appl Clin Med Phys. 2013;14(3):177‐191. 10.1120/jacmp.v14i3.4262 PMC5714427 23652257

[21] Mayyas E, Kim J, Kumar S, et al. A novel approach for evaluation of prostate deformation and associated dosimetric implications in IGRT of the prostate. Med Phys. 2014;41(9):091709. 25186384 10.1118/1.4893196

[22] Lim‐Reinders S, Keller BM, Al‐Ward S, Sahgal A, Kim A. Online adaptive radiation therapy. Int J Radiat Oncol Biol Phys. 2017;99(4):994‐1003. 28916139 10.1016/j.ijrobp.2017.04.023

[23] Green OL, Henke LE, Hugo GD. Practical clinical workflows for online and offline adaptive radiation therapy. Semin Radiat Oncol. 2019;29(3):219‐227. 31027639 10.1016/j.semradonc.2019.02.004 PMC6487881

[24] Langen KM, Willoughby TR, Meeks SL, et al. Observations on real‐time prostate gland motion using electromagnetic tracking. Int J Radiat Oncol Biol Phys. 2008;71(4):1084‐1090. 18280057 10.1016/j.ijrobp.2007.11.054

[25] Cai B, Laugeman E, Mazur TR, et al. Characterization of a prototype rapid kilovoltage x‐ray image guidance system designed for a ring shape radiation therapy unit. Med Phys. 2019;46(3):1355‐1370. 30675902 10.1002/mp.13396 PMC8188470

[26] Jarema T, Aland T. Using the iterative kV CBCT reconstruction on the Varian Halcyon linear accelerator for radiation therapy planning for pelvis patients. Phys Medica. 2019;68:112‐116. 10.1016/j.ejmp.2019.11.015 31783220

[27] Lim TY, Dragojević I, Hoffman D, Flores‐Martinez E, Kim GY. Characterization of the Halcyon TM multileaf collimator system. J Appl Clin Med Phys. 2019;20(4):106‐114. 30889312 10.1002/acm2.12568 PMC6448159

[28] Salembier C, Villeirs G, De Bari B, et al. ESTRO ACROP consensus guideline on CT‐ and MRI‐based target volume delineation for primary radiation therapy of localized prostate cancer. Radiother Oncol. 2018;127(1):49‐61. 29496279 10.1016/j.radonc.2018.01.014

[29] Steenbergen P, Haustermans K, Lerut E, et al. Prostate tumor delineation using multiparametric magnetic resonance imaging: Inter‐observer variability and pathology validation. Radiother Oncol. 2015;115(2):186‐190. 25935742 10.1016/j.radonc.2015.04.012

[30] Van SMA, Dinh CV, Van HPJ, et al. Contouring of prostate tumors on multiparametric MRI: Evaluation of clinical delineations in a multicenter radiotherapy trial. Radiother Oncol. 2018;128(2):321‐326. 29731160 10.1016/j.radonc.2018.04.015

[31] Lieberfarb ME, Schultz D, Whittington R, et al. Using PSA, biopsy Gleason score, clinical stage, and the percentage of positive biopsies to identify optimal candidates for prostate‐only radiation therapy. Int J Radiat Oncol. 2002;53(4):898‐903. 10.1016/s0360-3016(02)02812-2 12095555

[32] Gay HA, Barthold HJ, O’Meara E, et al. Pelvic normal tissue contouring guidelines for radiation therapy: a Radiation Therapy Oncology Group consensus panel atlas. Int J Radiat Oncol Biol Phys. 2012;83(3):e353‐e362. 22483697 10.1016/j.ijrobp.2012.01.023 PMC3904368

[33] Andreo P, Burns DT, Hohlfeld K, Huq MS, Kanai T, Laitano F, et al. Absorbed Dose Determination in External Beam Radiotherapy: An International Code of Practice for Dosimetry based on Standards of Absorbed Dose to Water. Technical Report Series No. 398. Iaea Trs‐398. 2006;2006(June):183.

[34] Michiels S, Poels K, Crijns W, et al. Volumetric modulated arc therapy of head‐and‐neck cancer on a fast‐rotating O‐ring linac: plan quality and delivery time comparison with a C‐arm linac. Radiother Oncol. 2008:128(3):479‐484. 10.1016/j.radonc.2018.04.021 29739713

[35] Lyman JT. Complication probability as assessed from dose‐volume histograms. Radiat Res Suppl. 1985;8:S13‐S19. 3867079

[36] Kutcher G, Burman C. Calculation of complication probability factors for non‐uniform normal tissue irradiation: the effective volume method. Int J Radiat Oncol Biol Phys. 1989;16:1623‐1630. 2722599 10.1016/0360-3016(89)90972-3

[37] Niemierko A. Reporting and analyzing dose distributions: a concept of equivalent uniform dose. Med Phys. 1997;24(1):103‐110. 9029544 10.1118/1.598063

[38] Michalski JM, Gay H, Jackson A, Tucker SL, Deasy JO. Radiation dose‐volume effects in radiation‐induced rectal injury. Int J Radiat Oncol Biol Phys. 2010;76(3):123‐129. 20171506 10.1016/j.ijrobp.2009.03.078 PMC3319467

[39] Kole TP, Tong M, Wu B, et al. Late urinary toxicity modeling after stereotactic body radiotherapy (SBRT) in the definitive treatment of localized prostate cancer. Acta Oncol (Madr). 2016;55(1):52‐58. 10.3109/0284186X.2015.1037011 PMC4986047 25972264

[40] Brand DH, Brüningk SC, Wilkins A, et al. Estimates of Alpha/Beta (α/β) ratios for individual late rectal toxicity endpoints: an analysis of the CHHiP trial. Int J Radiat Oncol Biol Phys. 2021;110(2):596‐608. 33412260 10.1016/j.ijrobp.2020.12.041 PMC8129972

[41] Benjamini Y, Hochberg Y. Controlling the false discovery rate: a practical and powerful approach to multiple testing. J R Stat Soc B. 1995;57(1):289‐300.

[42] Low DA, Harms WB, Mutic S, Purdy JA. A technique for the quantitative evaluation of dose distributions. Med Phys. 1998;25(5):656‐661. 9608475 10.1118/1.598248

[43] Miften M, Olch A, Mihailidis D, et al. Tolerance limits and methodologies for IMRT measurement‐based verification QA: Recommendations of AAPM Task Group. No. 218. Med Phys. 2018;45(4):e53‐83. 29443390 10.1002/mp.12810

[44] Pokhrel D, Tackett T, Stephen J, et al. Prostate SBRT using O‐Ring Halcyon Linac — Plan quality, delivery efficiency, and accuracy. J Appl Clin Med Phys. 2020;22(1):68‐75. 33340388 10.1002/acm2.13105 PMC7856496

[45] Woo JA, Chen LN, Bhagat A, et al. Clinical characteristics and management of late urinary symptom flare following stereotactic body radiation therapy for prostate cancer. Front Oncol. 2014;4:1‐10. 24904833 10.3389/fonc.2014.00122 PMC4033266

[46] De Roover R, Poels K, Michiels S. Validation and IMRT/VMAT delivery quality of a preconfigured fast‐rotating O‐ring linac system. Med Phys. 2019;46(1):328‐339. 30417523 10.1002/mp.13282

[47] Laugeman E, Heermann A, Hilliard J, et al. Comprehensive validation of halcyon 2.0 plans and the implementation of patient speci fic QA with multiple detector platforms. J Appl Clin Med Phys. 2020;(July 2019):1‐10. 10.1002/acm2.12881 PMC7386180 32368862

[48] Hansen CR, Crijns W, Hussein M, et al. RAdiotherapy treatment plannINg study guidelines (RATING): a framework for setting up and reporting on scientific treatment planning studies. Radiother Oncol. 2020;153:67‐78. 32976873 10.1016/j.radonc.2020.09.033

