# Supplementary material for: Automated treatment planning of prostate stereotactic body radiotherapy with focal boosting on a fast‐rotating O‐ring linac: Plan quality comparison with C‐arm linacs
Source: J Appl Clin Med Phys. 2021 Jul 28;22(9):59–72. doi: 10.1002/acm2.13345 (PMC8425873; doi:10.1002/acm2.13345)
Supplement: Supplementary file 1 — Supplementary Material [file ACM2-22-59-s001.pdf]

Supplemental Data

RATING score sheet

	RATING score sheet	Points	Applicable/ relevant	Answer yes
	**Questions for the Introduction**			
	The study aim formulated by research questions			
1	Does the study have a concise and precise study aim, defined with a restricted number of interconnected questions?	10		☑
	The motivation for the research questions			
2	Has relevant up to date literature been included to support the need for the current study?	5		☑
3	Does the study address an existing knowledge gap?	10		☑
	**Questions for Materials and Methods**			
4	Is the global study design adequate for answering the posed research questions?	10		☑
5	Is the global study design described in sufficient detail for others to interpret and reproduce the results?	5		☑
	Patient cohort			
6	Are the inclusion and exclusion criteria of the patient cohort described?	1	☑	☑
7	Is the clinical patient information of the cohort presented, including disease type, site(s) and clinical staging?	1	☑	☑
8	Is the included number of patients stated, explained and justified?	1	☑	☐
9	Has there been consideration of the need for ethical and/or legal approval for the study and if needed, is there a statement about this?	5		☐
	Imaging procedures			
10	Have the scanning parameters been reported in sufficient detail (image modalities, equipment model, slice thickness, voxel size, patient position (e.g. head first, supine, etc.) etc.)?	1	☑	☑
11	Has the applied immobilisation equipment been described, (e.g. vendor and type, standard settings, etc.) where relevant?	1	☑	☑
	Treatment machine and settings			
12	Have the treatment machine and relevant parameters been described with sufficient detail (model, beam energy, MLC, etc.)?	1	☑	☑
13	Have the monitor unit reference conditions been defined, where relevant?	1	☑	☑
	Definition of targets and OARs			
14	Has GTV definition been described in sufficient detail, with references if possible?	1	☑	☑
15	Has CTV definition been described in sufficient detail, with references if possible?	1	☑	☑
16	Has the establishment of PTVs (or alternatively robustness settings) been described in sufficient detail?	1	☑	☑
17	Have PTV sizes in the patient cohort been described?	1	☑	☑
18	Have OAR definitions been described in sufficient detail, with references if possible?	1	☑	☑
19	Have PRV margins been described in sufficient detail, with references if available?	1	☑	☑

	Treatment planning system and dose calculation			
20	Have all applied dose calculation algorithms been described in sufficient detail?	1	☑	☑
21	For any commercial software used, have the manufacturer, algorithms and specific versions been stated?	1	☑	☑
22	Have all relevant user parameters and settings in the TPS been reported, e.g. beams, dose grid, control point spacing?	1	☑	☑
23	Have all volumes been evaluated with the same software/methodology?	1	☑	☑
	Planning aims and optimisation			
24	Are clear planning aims defined, including imposed hard constraints and planning objectives (with or without soft constraints)?	5		☑
25	Has the ranking of planning objectives (priorities) been described?	5		☑
26	Is the dose prescription clearly defined?	10		☑
27	Is there a narrative description of the applied optimisation process, including the handling of all objectives with their ranking?	5		☑
28	If manual intervention during or after optimisation is allowed, has this been described?	1	☑	☑
	Bias mitigation			
29	Have enough study details been provided such that bias issues could be noted?	5		☑
30	Has bias been sufficiently mitigated to reliably answer the posed research question?	10		☑
	Plan acceptability – minor and major protocol deviations			
31	Was the procedure for assessment of plan acceptability well-described?	1	☑	☑
32	Was the procedure for assessment of minor and major protocol deviations well described?	1	☑	☐
	Plan (re-)normalisation for plan comparisons			
33	Has plan (re-)normalisation been described sufficiently?	1	☑	☑
	Dose-volume parameters for plan evaluation and comparison			
34	Have sufficiently comprehensive dose-volume parameters been used for plan evaluations and comparisons?	5		☑
	Population-mean DVHs			
35	Has the algorithm for creating population-mean/median DVHs been reported?	1	☐	☐
36	Have the definitions of confidence intervals been included?	1	☐	☐
	Plan evaluations by clinicians			
37	Have clinicians scored plans to assess quality?	1	☑	☐
38	Were plan comparisons by clinicians blinded?	1	☑	☐
	Predicted tumour control probability and normal tissue complication probabilities for plan evaluation and comparison			
39	Have any applied TCP models been described and referenced?	1	☐	☐
40	Have any applied NTCP models been described and referenced?	1	☑	☑
	Plan deliverability and complexity			
41	Have methods used to assess plan deliverability and complexity been described in sufficient detail?	1	☑	☑

	Composite plan quality metrics			
42	Is there a sufficient basis (e.g. in the literature) for any selected composite plan quality metrics?	1	☐	☐
43	Is there an adequate description of the calculation of the composite plan quality metrics?	1	☐	☐
	Planning and delivery times			
44	Has measurement of planning times been described in sufficient detail?	1	☑	☐
45	Has the establishment of delivery times been described in sufficient detail?	1	☑	☑
	Statistical analysis			
46	Have proper statistical methods been used and described in sufficient detail?	5		☑
47	In case of multiple testing for research questions, has this been handled appropriately?	1	☑	☑
**Questions for Results**				
48	Does the provided data contribute to (at least partly) answering all aspects of the research questions, e.g. plan acceptability, dosimetric quality, deliverability and planning and delivery times?	10		☑
	Dose distribution reporting			
49	Are complete summaries of the dose distributions in the patient cohort provided (low doses, high doses, OARs, PTV, patient, etc.)?	5		☑
50	Are tables and figures optimised to clearly present the results obtained?	1	☑	☑
51	Have the answers to the research questions been illustrated for an example patient by providing dose distributions, DVHs, etc.?	1	☑	☑
	Plan acceptability reporting – minor and major protocol deviations			
52	In case of treatment technique or planning technique comparisons, was plan acceptability reported separately for each technique?	1	☑	☑
53	Has plan acceptability been reported in sufficient detail: how many plans were acceptable, how many were not and for what reasons (e.g. violation of hard constraints, violation of soft constraints, other reasons)?	1	☑	☑
54	Was there adequate reporting of minor and major protocol deviations?	1	☑	☑
	Deliverability and complexity reporting			
55	Has the deliverability of the plans been adequately reported?	1	☑	☑
56	Have plan deliverability and complexity been investigated in sufficient detail in relation to the posed research questions?	1	☑	☑
	Planning and delivery times reporting			
57	Have planning and delivery times been adequately evaluated and reported?	1	☑	☑
	Patient-specific analyses reporting			
58	Is there sufficient description of inter-patient variations in the results presented?	1	☑	☐
59	Have outlier patients been reported and has any exclusion from population analyses been sufficiently motivated and explained?	1	☑	☑

	Statistical reporting			
60	Are the p-values reported appropriately?	1	☑	☑
61	Are there confidence intervals for the appropriate parameters?	1	☑	☐
**Questions for discussions**				
62	Is there an overall interpretation of the data presented in the Results section as to how the posed research questions are answered?	10		☑
	Comparison with literature			
63	Has the study been sufficiently discussed in the context of existing literature?	5		☐
	Clinical and statistical significance			
64	Does the discussion focus on statistically significant results?	1	☑	☑
65	Is the potential clinical significance of the results clearly discussed (assuming practical application would be feasible)?	5		☑
	Clinical applicability of the study			
66	Is future the clinical applicability sufficiently discussed?	1	☑	☑
	Study limitations			
67	Has the impact of the study limitations on the provided answers to the research questions been sufficiently discussed?	10		☑
	Future work			
68	Has the potential future work arising from the study been discussed?	1	☑	☑
**Questions for conclusions**				
69	Do the presented conclusions represent answers to the posed research questions?	5		☑
70	Are the conclusions fully supported by the results?	5		☑
71	Are the conclusions a fair summary of all results?	5		☑
**Questions for supplementary**				
	Supplementary materials			
72	Is the information presented in the supplementary material of sufficient relevance?	1	☑	☑
73	Is the presentation of the included information of sufficient quality, including readability?	1	☑	☑
74	Has sufficient underlying data been made available or a willingness to share data been indicated, within local data sharing restrictions?	5		☐
**RATING remarks**				
75	Is the RATING score added to the manuscript?	5		☑
76	Is the accompanying question table added to the cover letter or the supplementary material?	1	☑	☑

RATING score	90%		
**RATING fraction**	189	of	211

Supplemental Figures

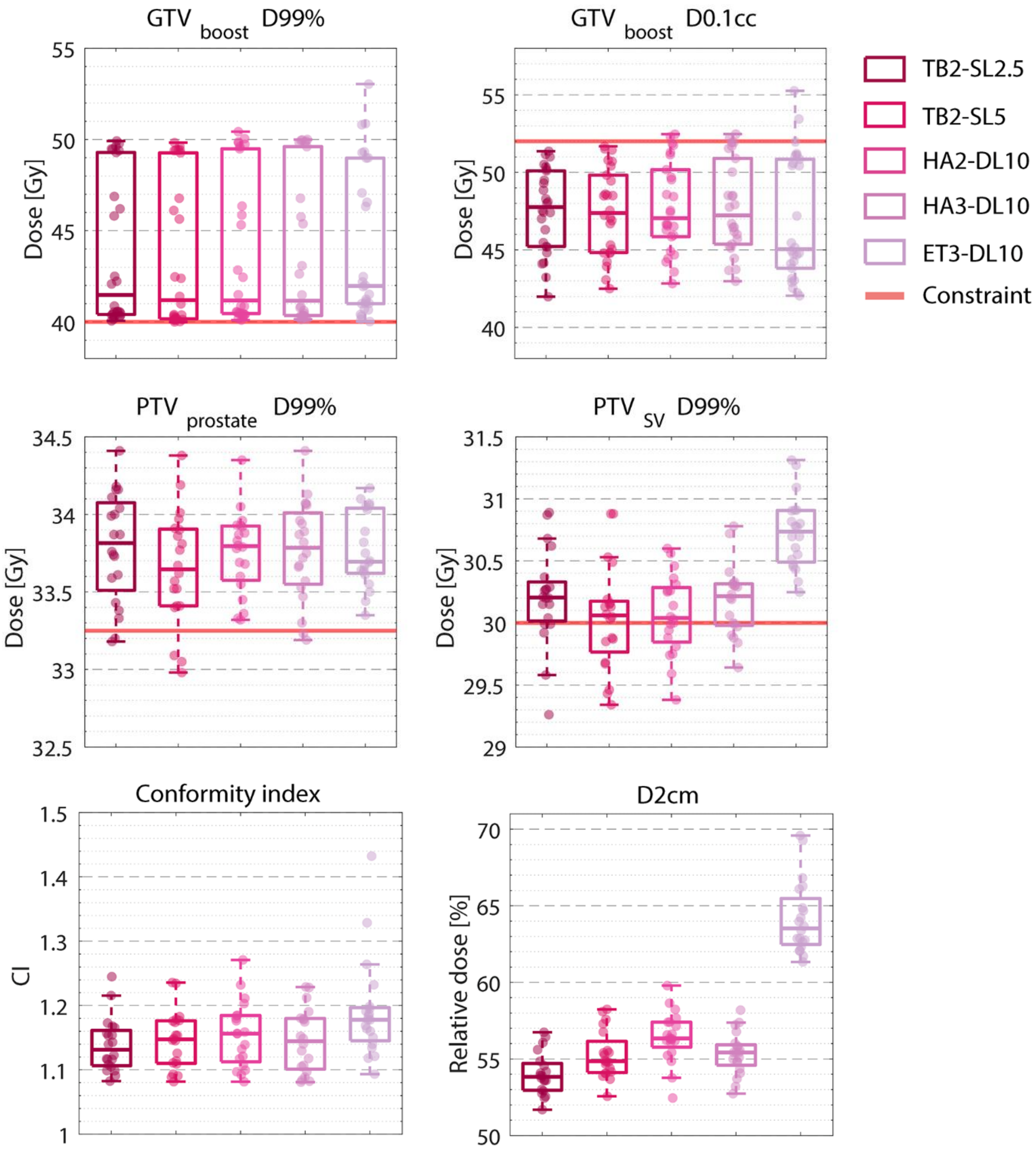

Supplemental Figure 1. Target coverage and high and intermediate dose spillage. Results are shown as boxplots over all patients for TB2-SL2.5 (outer left), TB2-SL5 (middle left), HA2-DL10 (middle), HA3-DL10 (middle right) and ET3-DL10 (outer right). The dots represent individual patients. Target volume dose prescriptions and constraints are indicated by a red solid line. The conformity index and dose at 2cm (D2cm) are calculated for $PTV_{prostate}$ and a prescribed dose of 35 Gy. The corresponding values are presented in Table 3.

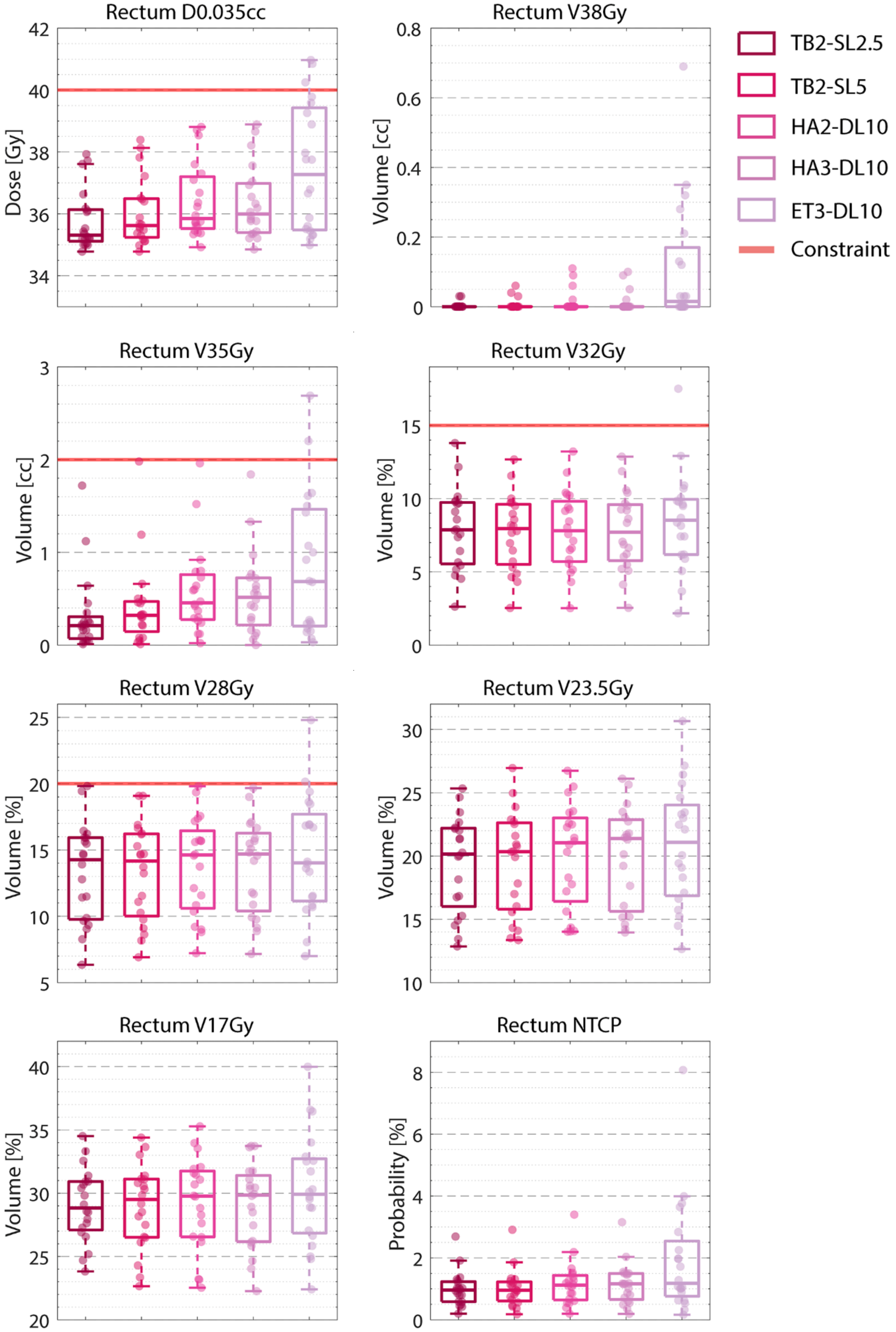

Supplemental Figure 2. Dose to the rectum. Results are shown as boxplots over all patients for TB2-SL2.5 (outer left), TB2-SL5 (middle left), HA2-DL10 (middle), HA3-DL10 (middle right) and ET3-DL10 (outer right). The dots represent individual patients. OAR dose constraints are indicated by a red solid line. The corresponding values are presented in Table 4.

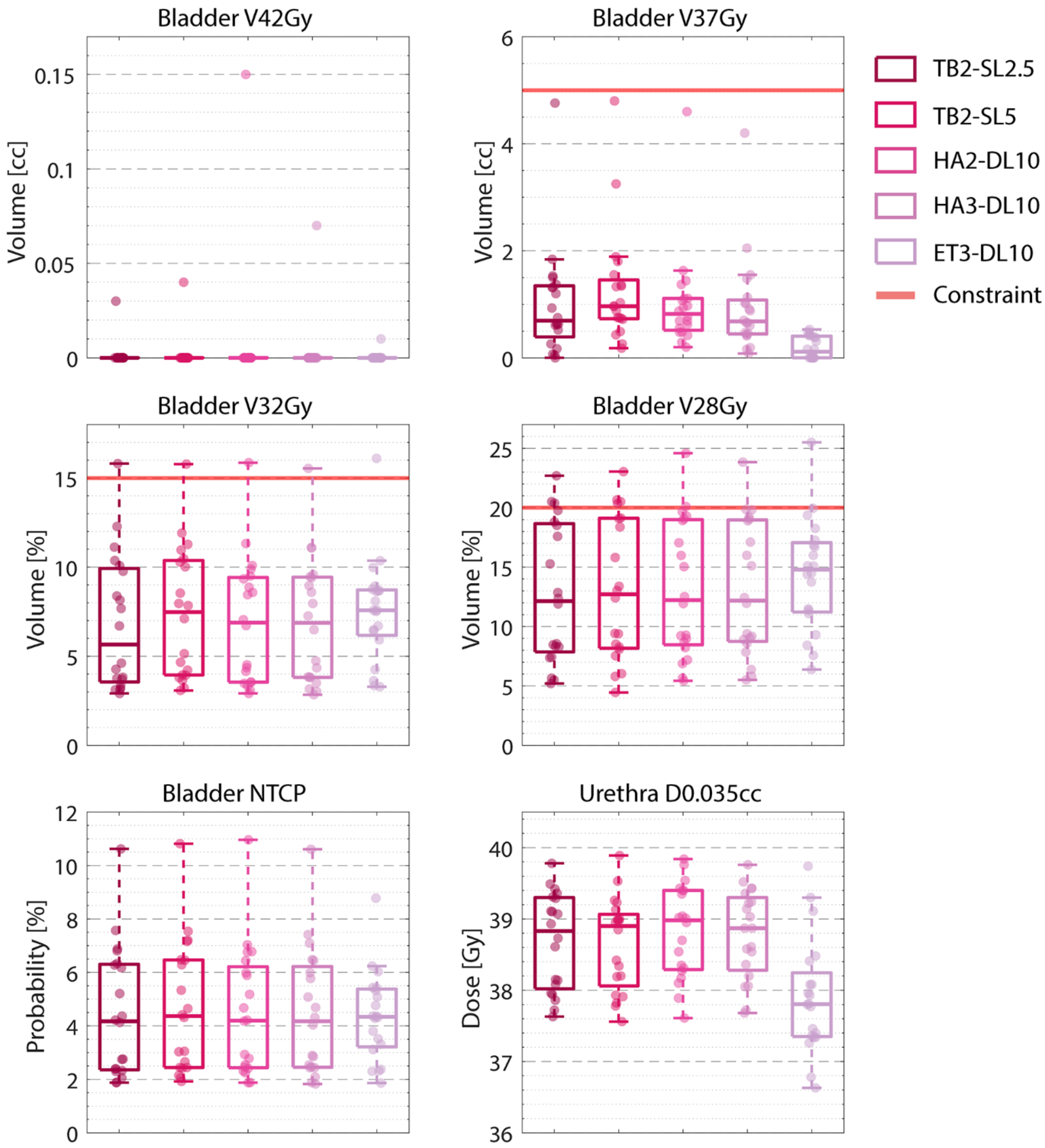

Supplemental Figure 3. Dose to the bladder and urethra. Results are shown as boxplots over all patients for TB2-SL2.5 (outer left), TB2-SL5 (middle left), HA2-DL10 (middle), HA3-DL10 (middle right) and ET3-DL10 (outer right). The dots represent individual patients. OAR dose constraints are indicated by a red solid line. The corresponding values are presented in Table 4.

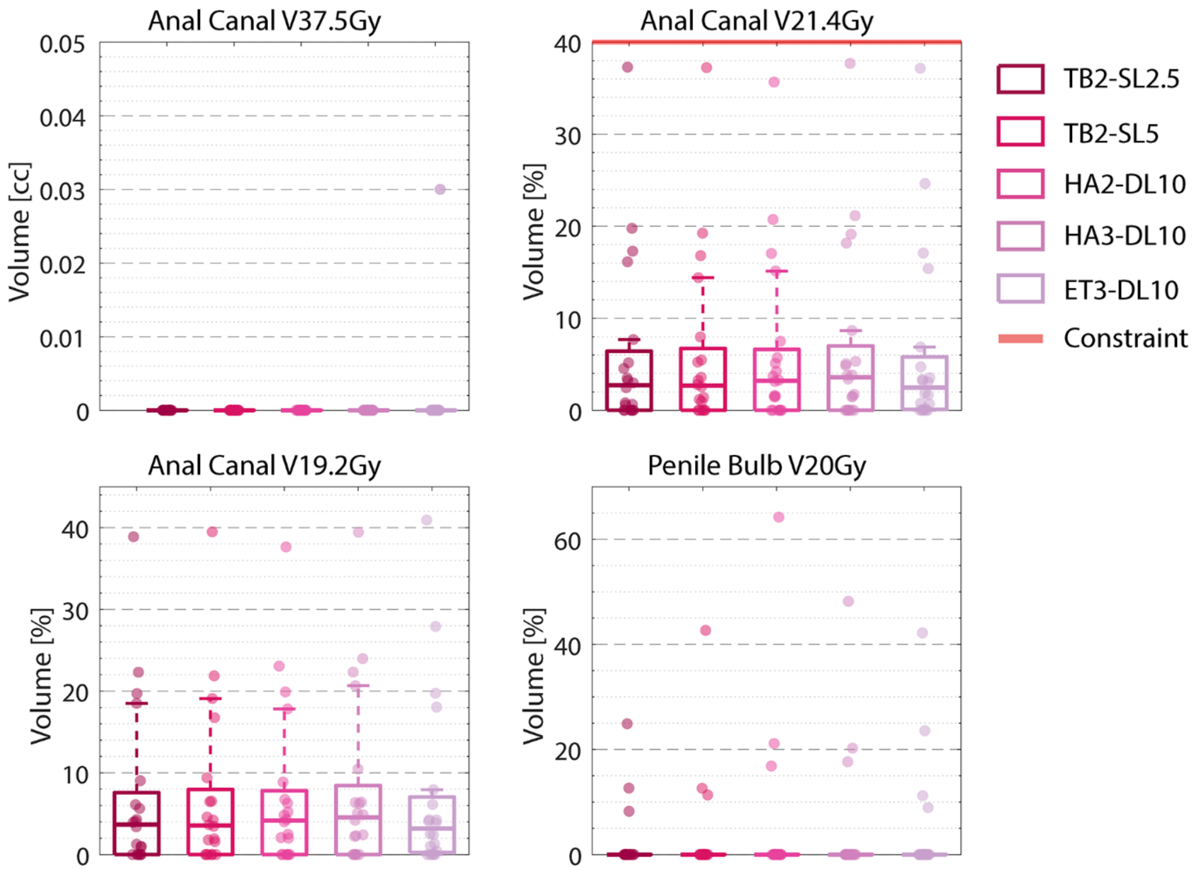

Supplemental Figure 4. Dose to the anal canal and penile bulb. Results are shown as boxplots over all patients for TB2-SL2.5 (outer left), TB2-SL5 (middle left), HA2-DL10 (middle), HA3-DL10 (middle right) and ET3-DL10 (outer right). The dots represent individual patients. OAR dose constraints are indicated by a red solid line. The corresponding values are presented in Table 4.

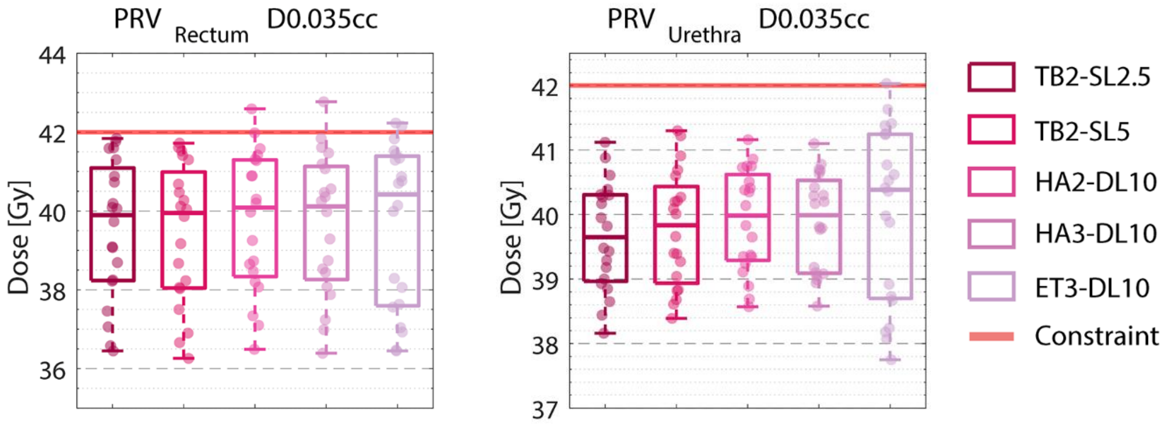

Supplemental Figure 5. Dose to the PRVs. Results are shown as boxplots over all patients for TB2-SL2.5 (outer left), TB2-SL5 (middle left), HA2-DL10 (middle), HA3-DL10 (middle right) and ET3-DL10 (outer right). The dots represent individual patients. OAR dose constraints are indicated by a red solid line. The corresponding values are presented in Table 4.

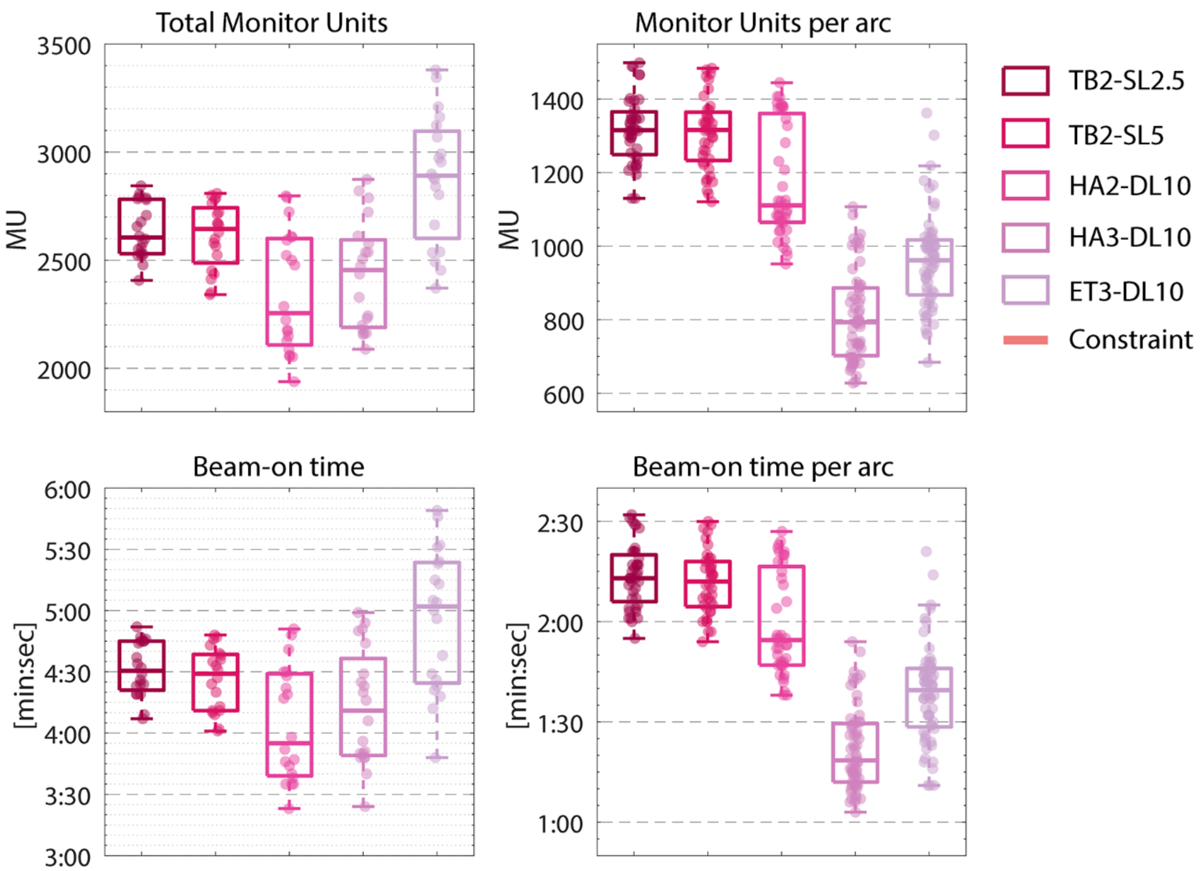

Supplemental Figure 6. Beam delivery parameters. Results are shown as boxplots over all patients for TB2-SL2.5 (outer left), TB2-SL5 (middle left), HA2-DL10 (middle), HA3-DL10 (middle right) and ET3-DL10 (outer right). The dots represent individual patients. The corresponding values are presented in Table 5.

Supplemental Tables

Supplemental Table 1 Imaging procedures and parameters used for patient simulation.

**Imaging modality**	**CT**	**mpMRI**		
Equipment model	Siemens Somatom Sensation Open	Siemens Magnetom 1.5T		
Sequence		T2w	DWI	DCE
Voxel size [mm²]	0.7 x 0.7	0.6 x 0.6	2.7 x 2.7	1.4 x 1.4
Slice thickness [mm]	1.0	3.0	4.0	4.0
Nominal energy [kVp]	125	-	-	-
Tube current [mAs]	AEC	-	-	-
TR [ms]	-	11250	9900	4.7
TE [ms]	-	124	67	1.6
Options	-	-	B = 0, 1000, 1400	-

Abbreviations: T2w = T2-weighted; DWI = diffusion weighted imaging; DCE = dynamic contrast enhanced; AEC = Automatic exposure contol; TR = repetition time; TE = echo time.

Supplemental Table 2 Initial optimization objectives that were used during manual treatment planning of TB2-SL2.5.

Structure	Objective type	Volume [%]	Dose [Gy]	Priority
**Target coverage objectives**				
GTV_{boost}	Upper	0.0	52.0	80
	Lower	100.0	40.0	200
$CTV_{prostate}$	Upper	0.0	52.0	80
	Lower	100.0	35.0	120
$PTV_{prostate}$	Upper	95.0	35.0	150
	Lower	95.0	35.0	150
	Lower	100.0	33.25	180
PTV_{SV}	Upper	0.0	35.0	80
	Lower	100.0	30.0	150
**OAR dose objectives**				
Rectum	Upper	0.0	36.0	180
	Upper	0.1	35.0	100
	Upper	20.0	28.0	150
	Upper	40.0	10.0	150
PRV_{rectum}	Upper	0.0	40.0	220
Bladder	Upper	20.0	27.5	150
Urethra	Upper	0.0	40.0	180
	Upper	20.0	39.0	120
$PRV_{urethra}$	Upper	0.0	40.0	220
Anal canal	Upper	39.5	21.4	150
	Upper	59.9	19.2	150
	Upper	0.0	37.5	150
Small bowel	Upper	0.0	34.5	180
Penile bulb	Upper	90.0	20.0	180
Femoral joint	Upper	5.0	27.0	150
**Auxiliary objectives**				
NS_GTV_{boost}+4mm	Upper	0.0	52.0	80
	Lower	100.0	40.0	200
NS_CTV_crop	Lower	100.0	35.5	220
NS_PTV_crop	Upper	0.0	38.5	120
	Lower	100.0	35.0	150
NS_PTVinRect	Upper	0.0	35.0	150
	Lower	100.0	33.25	150
NS_PTVminRect	Lower	100.0	35.0	150
NS_01_Ring_04	Upper	0.0	35.0	120
NS_05_Ring_15	Upper	0.0	28.0	120
NS_20_Ring_10	Upper	0.0	17.5	120

Supplemental Table 3 Initial clinical goals that were used as starting point for automated treatment planning of ET3-DL10

Structure	Clinical goal	Priority
**Target coverage goals**		
GTV_{boost}	$D_{99\%} \geq 40.0$ Gy	1
	$V_{40Gy} \geq 99.0\%$	1
	$D_{0.1cc} \leq 52.0$ Gy	4
$CTV_{prostate}$	$D_{99\%} \geq 35.0$ Gy	1
$PTV_{prostate}$	$D_{95\%} \geq 35.0$ Gy	2
	$D_{95\%} \leq 35.0$ Gy	2
	$D_{99\%} \geq 33.25$ Gy	1
CTV_{SV}	$D_{99\%} \geq 30.0$ Gy	1
PTV_{SV}	$D_{99\%} \geq 30.0$ Gy	1
	$D_{0.03cc} \leq 35.0$ Gy	4
**OAR goals**		
Rectum	$D_{0.03cc} \leq 40.0$ Gy	1
	$V_{38Gy} \leq 0.5$ cc (Var: ≤ 1.0 cc)	2
	$V_{35Gy} \leq 1.0$ cc (Var: ≤ 2.0 cc)	1
	$V_{32Gy} \leq 15\%$	3
	$V_{28Gy} \leq 20\%$	3
	$V_{23.5Gy} \leq 50\%$	3
	$V_{17Gy} \leq 75\%$	3
	$V_{10Gy} \leq 40\%$	4
PRV_{rectum}	$D_{0.03cc} \leq 42.0$ Gy	1
	$D_{0.03cc} \leq 40.0$ Gy	3
Bladder	$V_{42Gy} \leq 0.1$ cc (Var: ≤ 1.0 cc)	2
	$V_{37Gy} \leq 3.0$ cc (Var: ≤ 5.0 cc)	2
	$V_{32Gy} \leq 15\%$	2
	$V_{28Gy} \leq 20\%$	1
Urethra	$D_{0.03cc} \leq 40.0$ Gy (Var: ≤ 42.0 Gy)	1
$PRV_{urethra}$	$D_{0.03cc} \leq 42.0$ Gy	1
	$D_{0.03cc} \leq 40.0$ Gy (Var: ≤ 42.0 Gy)	3
Anal canal	$D_{mean} \leq 17.0$ Gy	3
	$V_{37.85Gy} \leq 1.0$ cc	3
	$V_{23.4Gy} \leq 40.0\%$	3
	$V_{19.7Gy} \leq 60.0\%$	3
Penile bulb	$V_{20Gy} \leq 50.0\%$ (Var: $\leq 90.0\%$)	3
Femoral joint	$V_{28Gy} \leq 3.0\%$ (Var: $\leq 5.0\%$)	3

(continued on next page)

Supplemental Table 3 *(continued)*		
**Auxilliary goals**		
NS_PTV_crop	$D_{99\%} \geq 35.0$ Gy	3
	$D_{0.03cc} \leq 38.5$ Gy (Var: ≤ 39.5 Gy)	3

Abbreviations: Var: allowed variability of the clinical goal that does not result in a protocol violation.
The priority of each goal is specified on a scale from 1 (most important) to 4 (less important).

Auxiliary structures:

- NS_GTV_{boost}+4mm is defined as GTV_{boost} with a 4 mm isotropic margin excluding all OARs and PRVs.
- NS_CTV_crop is defined as $CTV_{prostate}$ with a 1 mm isotropic inner margin excluding GTV_{boost} with a 4 mm isotropic margin.
- NS_PTV_crop is defined as $PTV_{prostate}$ excluding GTV_{boost} with a 7 mm isotropic margin and also excluding the rectum and bladder.
- NS_PTVinRect is defined as the overlap between $PTV_{prostate}$ and the rectum.
- NS_PTVminRect is defined as $PTV_{prostate}$ excluding the rectum.
- NS_01_Ring_04 is a wall structure starting at 1 mm outside of $PTV_{prostate}$ and PTV_{SV} and is 4 mm wide.
- NS_05_Ring_15 is a wall structure starting at 5 mm outside of $PTV_{prostate}$ and PTV_{SV} and is 15 mm wide.
- NS_20_Ring_10 is a wall structure starting at 20 mm outside of $PTV_{prostate}$ and PTV_{SV} and is 10 mm wide.

Supplemental Table 4 Pairwise dosimetric comparison between all planning techniques.

	TB2-SL5 vs TB2-SL2.5 Median (range)	HA2-DL10 vs TB2-SL2.5 Median (range)	HA3-DL10 vs TB2-SL2.5 Median (range)	ET3-DL10 vs TB2-SL2.5 Median (range)	HA2-DL10 vs TB2-SL5 Median (range)	HA3-DL10 vs TB2-SL5 Median (range)	ET3-DL10 vs TB2-SL5 Median (range)	HA3-DL10 vs HA2-DL10 Median (range)	ET3-DL10 vs HA2-DL10 Median (range)	ET3-DL10 vs HA3-DL10 Median (range)
**Target coverage**										
GTV_{boost}										
$D_{99\%}$ [Gy]	-0.1 (-0.7, 0.2)	0.0 (-0.6, 0.7)	0.0 (-0.6, 0.9)	0.2 (-1.0, 3.6)	0.2 (-0.4, 0.6)	0.2 (-0.4, 0.7)	0.4 (-0.5, 3.7)	0.0 (-0.4, 0.4)	0.3 (-1.1, 3.4)	0.3 (-1.1, 3.1)
$D_{0.1cc}$ [Gy]	0.0 (-2.4, 0.9)	0.2 (-1.9, 1.5)	0.4 (-1.5, 1.8)	-0.5 (-4.9, 4.3)	0.3 (-1.1, 1.6)	0.4 (-1.9, 2.1)	-0.4 (-4.4, 4.0)	0.1 (-1.3, 1.8)	-1.1 (-4.4, 3.8)	-1.0 (-4.8, 3.4)
$PTV_{prostate}$										
$D_{99\%}$ [Gy]	-0.2 (-0.3, 0.1)	-0.1 (-0.3, 0.3)	0.0 (-0.2, 0.2)	-0.1 (-0.6, 0.9)	0.1 (-0.3, 0.4)	0.1 (-0.1, 0.5)	0.1 (-0.6, 1.1)	-0.2 (-0.2, 0.2)	0.0 (-0.4, 0.8)	-0.1 (-0.5, 0.9)
PTV_{SV}										
$D_{99\%}$ [Gy]	-0.2 (-0.6, 0.3)	-0.2 (-0.4, 0.1)	0.0 (-0.6, 0.4)	0.5 (-0.2, 1.2)	-0.1 (-0.4, 0.6)	0.1 (-0.2, 0.8)	0.7 (-0.3, 1.4)	0.1 (-0.3, 0.4)	0.7 (0.1, 1.2)	0.6 (-0.1, 1.0)
**Dose spillage**										
CI	0.01 (-0.01, 0.03)	0.02 (-0.03, 0.08)	0.00 (-0.02, 0.06)	0.04 (-0.08, 0.26)	0.01 (-0.05, 0.08)	-0.01 (-0.03, 0.05)	0.03 (-0.09, 0.26)	-0.01 (-0.06, 0.04)	0.02 (-0.11, 0.25)	0.03 (-0.07, 0.23)
D2cm [%]	1.2 (0.7, 2.4)	2.5 (0.7, 5.1)	1.4 (-0.9, 3.1)	9.8 (6.0, 15.7)	1.1 (-0.6, 4.2)	0.1 (-2.8, 1.6)	8.5 (4.7, 14.8)	-1.0 (-3.3, 0.4)	7.1 (4.5, 13.2)	8.7 (4.6, 14.3)
**OAR dose**										
Rectum										
$D_{0.035cc}$ [Gy]	0.3 (0.0, 0.6)	0.6 (0.1, 1.3)	0.6 (0.1, 1.3)	1.7 (0.1, 3.8)	0.4 (-0.2, 0.8)	0.3 (-0.1, 0.8)	1.4 (-0.3, 3.6)	-0.1 (-0.4, 0.4)	1.0 (-0.6, 3.4)	0.8 (-0.8, 3.3)
V_{38Gy} [cc]	0.00 (0.00, 0.04)	0.00 (0.00, 0.09)	0.00 (0.00, 0.10)	0.02 (0.00, 0.66)	0.00 (0.00, 0.05)	0.00 (0.00, 0.06)	0.02 (0.00, 0.63)	0.00 (-0.02, 0.01)	0.02 (0.00, 0.58)	0.02 (0.00, 0.06)
V_{35Gy} [cc]	0.11 (0.00, 0.26)	0.25 (0.01, 0.54)	0.20 (-0.01, 0.52)	0.36 (-0.41, 1.57)	0.16 (-0.02, 0.40)	0.14 (-0.14, 0.40)	0.30 (-0.43, 1.53)	-0.02 (-0.19, 0.12)	0.07 (-0.69, 1.31)	0.12 (-0.74, 1.45)
V_{32Gy} [%]	-0.2 (-1.1, 0.6)	-0.1 (-1.5, 0.9)	0.0 (-1.5, 0.8)	0.3 (-1.5, 3.8)	0.2 (-1.0, 0.8)	0.2 (-1.1, 1.1)	0.4 (-1.4, 4.8)	0.0 (-1.2, 0.6)	0.5 (-2.0, 4.3)	0.5 (-1.2, 4.6)
V_{28Gy} [%]	-0.1 (-1.0, 0.6)	0.5 (-0.2, 1.3)	0.3 (-0.7, 2.1)	1.1 (-2.1, 5.0)	0.5 (-0.6, 1.1)	0.4 (-1.2, 1.5)	0.7 (-1.9, 5.7)	-0.1 (-2.1, 1.3)	0.3 (-2.2, 5.0)	0.0 (-1.5, 5.1)

(continued on next page)

Suplementary Table 4 *(continued)*

	TB2-SL5 vs TB2-SL2.5 Median (range)	HA2-DL10 vs TB2-SL2.5 Median (range)	HA3-DL10 vs TB2-SL2.5 Median (range)	ET3-DL10 vs TB2-SL2.5 Median (range)	HA2-DL10 vs TB2-SL5 Median (range)	HA3-DL10 vs TB2-SL5 Median (range)	ET3-DL10 vs TB2-SL5 Median (range)	HA3-DL10 vs HA2-DL10 Median (range)	ET3-DL10 vs HA2-DL10 Median (range)	ET3-DL10 vs HA3-DL10 Median (range)
Rectum										
$V_{23.5Gy}$ [%]	0.0 (-1.0, 2.3)	0.5 (-0.9, 2.1)	0.6 (-1.2, 2.5)	1.7 (-2.8, 5.3)	0.6 (-0.4, 1.4)	0.4 (-0.8, 2.2)	1.0 (-2.2, 5.7)	-0.3 (-1.5, 1.6)	0.8 (-3.2, 5.2)	0.6 (-2.3, 5.0)
$V_{20.5Gy}$ [%]	0.0 (-1.5, 1.2)	0.2 (-1.6, 2.0)	0.4 (-1.9, 1.8)	1.3 (-3.0, 5.7)	0.5 (-1.2, 1.6)	0.3 (-1.0, 2.2)	0.8 (-2.5, 5.8)	-0.2 (-1.7, 1.5)	1.0 (-3.4, 5.5)	0.6 (-2.1, 5.3)
V_{17Gy} [%]	0.0 (-2.1, 1.0)	0.2 (-2.2, 2.7)	-0.1 (-2.4, 1.8)	0.7 (-2.5, 7.4)	0.2 (-2.3, -2.2)	0.2 (-2.0, 2.0)	1.1 (-1.9, 6.6)	-0.3 (-1.6, 1.5)	1.0 (-3.0, 6.5)	0.7 (-1.7, 6.3)
PRV_{rectum}										
$D_{0.035cc}$ [Gy]	-0.1 (-0.5, 0.1)	0.2 (-0.6, 1.0)	0.1 (-0.7, 1.2)	0.0 (-1.5, 2.0)	0.2 (-0.2, 0.9)	0.2 (-0.2, 1.1)	0.2 (-1.1, 2.0)	-0.1 (-0.4, 0.2)	-0.1 (-1.3, 1.3)	-0.2 (-1.5, 1.8)
Bladder										
V_{42Gy} [cc]	0.00 (0.00, 0.01)	0.00 (0.00, 0.12)	0.00 (0.00, 0.04)	0.00 (-0.02, 0.00)	0.00 (0.00, 0.11)	0.00 (0.00, 0.03)	0.00 (-0.03, 0.00)	0.00 (-0.08, 0.00)	0.00 (-0.14, 0.00)	0.00 (-0.06, 0.00)
V_{37Gy} [cc]	0.21 (-0.17, 1.75)	-0.02 (-0.73, 0.52)	0.01 (-0.84, 0.74)	-0.61 (-4.27, 0.00)	-0.19 (-2.48, 0.29)	-0.30 (-2.59, 0.25)	-0.77 (-4.31, -0.18)	-0.05 (-0.50, 0.42)	-0.69 (-4.11, -0.20)	-0.57 (-3.71, -0.08)
V_{32Gy} [%]	0.2 (-0.4, 7.6)	-0.1 (-1.0, 6.1)	-0.3 (-1.8, 6.0)	0.5 (-2.5, 5.4)	-0.4 (-1.5, 0.6)	-0.3 (-2.1, 1.0)	0.3 (-2.7, 3.9)	0.0 (-0.9, 1.0)	0.4 (-1.5, 4.0)	0.5 (-2.5, 3.8)
V_{28Gy} [%]	0.5 (-1.1, 1.1)	0.4 (-1.1, 2.2)	0.4 (-1.2, 2.3)	1.9 (-2.9, 6.2)	-0.1 (-1.4, 1.7)	0.0 (-1.4, 1.7)	1.5 (-3.5, 6.3)	0.0 (-0.8, 0.7)	1.0 (-2.8, 5.7)	1.0 (-3.0, 5.6)
Urethra										
$D_{0.035cc}$ [Gy]	0.0 (-0.4, 0.2)	0.1 (-0.6, 0.8)	0.1 (-0.4, 0.8)	-0.8 (-1.5, 0.3)	0.2 (-0.6, 1.1)	0.1 (-0.2, 1.1)	-0.9 (-1.5, 0.3)	-0.1 (-0.7, 0.5)	-1.0 (-2.0, 0.2)	-1.1 (-1.5, 0.3)
$PRV_{urethra}$										
$D_{0.035cc}$ [Gy]	0.0 (-0.2, 1.4)	0.2 (-0.7, 1.5)	0.1 (-0.3, 1.4)	0.6 (-1.6, 1.3)	0.2 (-0.7, 0.5)	0.1 (-0.6, 0.6)	0.3 (-1.5, 1.4)	-0.1 (-0.4, 1.1)	0.2 (-1.7, 1.4)	0.2 (-1.6, 1.4)
Anal canal										
$V_{37.85Gy}$ [cc]	-	-	-	-	-	-	-	-	-	-
$V_{21.4Gy}$ [%]	0.0 (-1.7, 0.8)	0.2 (-4.6, 3.4)	0.5 (-0.6, 3.9)	0.0 (-2.1, 4.9)	0.1 (-4.1, 3.9)	0.3 (-0.2, 4.4)	0.0 (-2.4, 5.4)	0.0 (-0.4, 4.0)	0.0 (-3.7, 9.5)	-0.2 (-4.1, 5.5)
$V_{19.2Gy}$ [%]	0.0 (-1.8, 1.0)	0.3 (-4.5, 3.4)	0.7 (-0.1, 4.3)	0.0 (-2.2, 5.6)	0.1 (-4.1, 4.0)	0.4 (-0.2, 4.9)	0.0 (-2.6, 6.0)	0.2 (-0.4, 4.5)	0.0 (-3.3, 10.1)	-0.1 (-4.2, 5.6)

(continued on next page)

Suplementary Table 4 *(continued)*

	TB2-SL5 vs TB2-SL2.5	HA2-DL10 vs TB2-SL2.5	HA3-DL10 vs TB2-SL2.5	ET3-DL10 vs TB2-SL2.5	HA2-DL10 vs TB2-SL5	HA3-DL10 vs TB2-SL5	ET3-DL10 vs TB2-SL5	HA3-DL10 vs HA2-DL10	ET3-DL10 vs HA2-DL10	ET3-DL10 vs HA3-DL10
	Median (range)	Median (range)	Median (range)	Median (range)	Median (range)	Median (range)	Median (range)	Median (range)	Median (range)	Median (range)
Penile bulb										
V_{20Gy} [%]	0.0 (-0.0, 17.8)	0.0 (0.0, 39.3)	0.0 (0.0, 23.3)	0.0 (0.0, 17.3)	0.0 (0.0, 21.6)	0.0 (0.0, 7.6)	0.0 (-0.5, 11.0)	0.0 (-16.0, 0.8)	0.0 (-22.0, 8.9)	0.0 (-6.5, 8.9)
Femoral head & neck										
V_{28Gy} [%]	-	-	-	-	-	-	-	-	-	-
**NTCP**										
Rectum [%]	0.0 (-0.1, 0.2)	0.1 (0.0, 0.7)	0.1 (-0.1, 0.5)	0.5 (-0.2, 5.4)	0.1 (0.0, 0.5)	0.1 (0.0, 0.5)	0.5 (-0.2, 5.2)	0.0 (-0.2, 0.2)	0.4 (-0.2, 4.7)	0.4 (-0.4, 4.9)
Bladder [%]	0.2 (0.0, 0.4)	0.0 (-0.5, 0.4)	0.1 (-0.6, 0.6)	0.0 (-1.8, 1.9)	-0.1 (-0.6, 0.3)	-0.2 (-0.7, 0.3)	-0.3 (-2.0, 1.9)	0.0 (-0.4, 0.6)	-0.2 (-2.2, 1.9)	-0.2 (-2.1, 1.7)

Bold values indicate a statistically significant difference for the two-sided Wilcoxon's matched-pairs signed-rank test after performing the Benjamini-Hochberg procedure to correct for multiple comparisons at significance level 0.05. The corresponding p-values are presented in Supplemental Table 5.

Supplemental Table 5 Statistical analysis.

	Kruskal-Wallis omnibus test	Wilcoxon's matched-pairs signed rank test[a]									
		TB2-SL5 vs TB2-SL2.5	HA2-DL10 vs TB2-SL2.5	HA3-DL10 vs TB2-SL2.5	ET3-DL10 vs TB2-SL2.5	HA2-DL10 vs TB2-SL5	HA3-DL10 vs TB2-SL5	ET3-DL10 vs TB2-SL5	HA3-DL10 vs HA2-DL10	ET3-DL10 vs HA2-DL10	ET3-DL10 vs HA3-DL10
	p-value	p-value	p-value	p-value	p-value	p-value	p-value	p-value	p-value	p-value	p-value
**Target coverage**											
GTV_{boost}											
$D_{99\%}$ [Gy]	0,631	-	-	-	-	-	-	-	-	-	-
$D_{0.1cc}$ [Gy]	0,748	-	-	-	-	-	-	-	-	-	-
$PTV_{prostate}$											
$D_{99\%}$ [Gy]	0,749	-	-	-	-	-	-	-	-	-	-
PTV_{SV}											
$D_{99\%}$ [Gy]	**< 0.001**	**0,002**	**0,002**	0,735	**< 0.001**	0,978	0,099	**< 0.001**	**0,008**	**< 0.001**	**< 0.001**
**Dose spillage**											
CI	0,058	-	-	-	-	-	-	-	-	-	-
D2cm [%]	**< 0.001**	**< 0.001**	**< 0.001**	**< 0.001**	**< 0.001**	**< 0.001**	0,43	**< 0.001**	**< 0.001**	**< 0.001**	**< 0.001**
**OAR dose**											
Rectum											
$D_{0.035cc}$ [Gy]	0,015	-	-	-	-	-	-	-	-	-	-
V_{38Gy} [cc]	**0,008**	0,5	0,125	0,125	**0,002**	0,125	0,125	**0,002**	0,75	**0,002**	**0,002**
V_{35Gy} [cc]	0,026	-	-	-	-	-	-	-	-	-	-
V_{32Gy} [%]	0,929	-	-	-	-	-	-	-	-	-	-
V_{28Gy} [%]	0,844	-	-	-	-	-	-	-	-	-	-
$V_{23.5Gy}$ [%]	0,823	-	-	-	-	-	-	-	-	-	-
$V_{20.5Gy}$ [%]	0,92	-	-	-	-	-	-	-	-	-	-

(continued on next page)

Supplemental Table 5 *(continued)*

	Kruskal-Wallis omnibus test	Wilcoxon's matched-pairs signed rank test[a]									
		TB2-SL5 vs TB2-SL2.5	HA2-DL10 vs TB2-SL2.5	HA3-DL10 vs TB2-SL2.5	ET3-DL10 vs TB2-SL2.5	HA2-DL10 vs TB2-SL5	HA3-DL10 vs TB2-SL5	ET3-DL10 vs TB2-SL5	HA3-DL10 vs HA2-DL10	ET3-DL10 vs HA2-DL10	ET3-DL10 vs HA3-DL10
	p-value	p-value	p-value	p-value	p-value	p-value	p-value	p-value	p-value	p-value	p-value
Rectum											
V_{17Gy} [%]	0,913	-	-	-	-	-	-	-	-	-	-
PRV_{rectum}											
$D_{0.035cc}$ [Gy]	0,977	-	-	-	-	-	-	-	-	-	-
Bladder											
V_{42Gy} [cc]	1	-	-	-	-	-	-	-	-	-	-
V_{37Gy} [cc]	**< 0.001**	**< 0.001**	1	0,749	**< 0.001**	**0,003**	**0,002**	**< 0.001**	0,22	**< 0.001**	**< 0.001**
V_{32Gy} [%]	0,906	-	-	-	-	-	-	-	-	-	-
V_{28Gy} [%]	0,911	-	-	-	-	-	-	-	-	-	-
Urethra											
$D_{0.035cc}$ [Gy]	**0,001**	0,578	0,078	0.220	**< 0.001**	0,043	0,121	**< 0.001**	0,492	**< 0.001**	**< 0.001**
$PRV_{urethra}$											
$D_{0.035cc}$ [Gy]	0,775	-	-	-	-	-	-	-	-	-	-
Anal canal											
$V_{37.85Gy}$ [cc]	0,089	-	-	-	-	-	-	-	-	-	-
$V_{21.4Gy}$ [%]	0,989	-	-	-	-	-	-	-	-	-	-
$V_{19.2Gy}$ [%]	0,988	-	-	-	-	-	-	-	-	-	-
Penile bulb											
V_{20Gy} [%]	0,993	-	-	-	-	-	-	-	-	-	-

(continued on next page)

Supplemental Table 5 *(continued)*

	Kruskal-Wallis omnibus test	Wilcoxon's matched-pairs signed rank test[a]									
		TB2-SL5 vs TB2-SL2.5	HA2-DL10 vs TB2-SL2.5	HA3-DL10 vs TB2-SL2.5	ET3-DL10 vs TB2-SL2.5	HA2-DL10 vs TB2-SL5	HA3-DL10 vs TB2-SL5	ET3-DL10 vs TB2-SL5	HA3-DL10 vs HA2-DL10	ET3-DL10 vs HA2-DL10	ET3-DL10 vs HA3-DL10
	p-value	p-value	p-value	p-value	p-value	p-value	p-value	p-value	p-value	p-value	p-value
Femoral head & neck											
V_{28Gy} [%]	1	-	-	-	-	-	-	-	-	-	-
**NTCP**											
Rectum [%]	0,341	-	-	-	-	-	-	-	-	-	-
Bladder [%]	0,989	-	-	-	-	-	-	-	-	-	-

Bold values indicate a statistically significant p-value after performing the Benjamini-Hochberg procedure to correct for multiple comparisons at significance level 0.05.

[a] Wilcoxon's matched-pairs signed rank test was only performed for plan quality metrics that showed a statistically significant p-value for the Kruskal-Wallis omnibus test.
