# Supplementary material for: Automated treatment planning of prostate stereotactic body radiotherapy with focal boosting on a fast‐rotating O‐ring linac: Plan quality comparison with C‐arm linacs
Source: J Appl Clin Med Phys. 2021 Jul 28;22(9):59–72. doi: 10.1002/acm2.13345 (PMC8425873; doi:10.1002/acm2.13345)
Supplement: Supplementary file 2 — Supplementary Material [file ACM2-22-59-s002.pdf]

Supplemental Methods and materials

Normal tissue complication probability calculation

Normal tissue complication probability (NTCP) for the rectum and bladder were calculated using the Lyman-Kutcher-Burman (LKB) model with Niemierko's equivalent uniform dose [1–3]. Rectum NTCP was calculated using the best estimate QUANTEC parameters for late grade ≥2 rectal toxicity or bleeding [4]. Bladder NTCP was calculated using the parameters derived by Kole et al. for late urinary symptom flare after prostate SBRT in 5 fractions [5].

First, the cumulative dose volume histograms (DVHs) of the rectum and the bladder were extracted from the treatment planning system and converted into relative volume differential DVHs using an in-house Matlab script (MathWorks, Natick, MA).

For the rectum, DVH dose bins were converted to the equivalent dose in 2 Gy fractions according to,

$$EQD_2 = D\frac{\alpha/\beta + d}{\alpha/\beta + 2}$$

with D the total dose and d the dose per fraction. An α/β ratio of 3 Gy was used as recommended by the QUANTEC study and the late rectal toxicity endpoint analysis of the CHHiP trial [4,6].

For the bladder, biological effective dose correction was not necessary as the studied fractionation scheme (5 fractions) was identical to the one used for modelling by Kole et al. [5].

Next, the (corrected) differential DVHs were used to calculate Niemierko's equivalent uniform dose (EUD):

$$EUD = \left(\sum_i D_i^{\frac{1}{n}} V_i\right)^n$$

with V_i the relative differential volume of dose bin i that receives a dose D_i and n the volume effect parameter [3]. The dose D_i is calculated as the average of the lower and upper dose limit of the dose bin.

The NTCP was then calculated according to:

$$NTCP = \frac{1}{\sqrt{2\pi}}\int_{-\infty}^{t} e^{-\frac{x^2}{2}}dx$$

with

$$t = \frac{EUD - TD_{50}}{m \cdot TD_{50}}$$

where TD_{50} is the dose that will result in 50% of complication in a uniformly irradiated tissue and m is inversely proportional to the slope of the steepest point on the NTCP versus EUD curve.

The NTCP parameters were $n = 0.09, m = 0.13$ and $TD_{50} = 76.9\ Gy$ for the rectum NTCP [4] and $n = 0.03, m = 0.19$ and $TD_{50} = 38.7\ Gy$ for the bladder NTCP [5].

References

1. Lyman JT. Complication Probability as Assessed from Dose-Volume Histograms. Radiat Res Suppl. 1985;8:S13–9.
2. Kutcher G, Burman C. Calculation of complication probability factors for non-uniform normal tissue irradiation: the effective volume method. Int J Radiat Oncol Biol Phys. 1989;16:1623–30.
3. Niemierko A. Reporting and analyzing dose distributions: A concept of equivalent uniform dose. Med Phys. 1997;24(1):103–10.
4. Michalski JM, Gay H, Jackson A, Tucker SL, Deasy JO. Radiation Dose-Volume Effects in Radiation-Induced Rectal Injury. Int J Radiat Oncol Biol Phys. 2010;76(3 SUPPL.):123–9.
5. Kole TP, Tong M, Wu B, Lei S, Obayomi-Davies O, Chen LN, et al. Late urinary toxicity modeling after stereotactic body radiotherapy (SBRT) in the definitive treatment of localized prostate cancer. Acta Oncol (Madr). 2016;55(1):52–8.
6. Brand DH, Brüningk SC, Wilkins A, Fernandez K, Naismith O, Gao A, et al. Estimates of Alpha/Beta (α/β) Ratios for Individual Late Rectal Toxicity Endpoints: An Analysis of the CHHiP trial. Int J Radiat Oncol Biol Phys. 2021;
